# Enhanced Longevity by Ibuprofen, Conserved in Multiple Species, Occurs in Yeast through Inhibition of Tryptophan Import

**DOI:** 10.1371/journal.pgen.1004860

**Published:** 2014-12-18

**Authors:** Chong He, Scott K. Tsuchiyama, Quynh T. Nguyen, Ekaterina N. Plyusnina, Samuel R. Terrill, Sarah Sahibzada, Bhumil Patel, Alena R. Faulkner, Mikhail V. Shaposhnikov, Ruilin Tian, Mitsuhiro Tsuchiya, Matt Kaeberlein, Alexey A. Moskalev, Brian K. Kennedy, Michael Polymenis

**Affiliations:** 1 Buck Institute for Research on Aging, Novato, California, United States of America; 2 Department of Biochemistry and Biophysics, Texas A&M University, College Station, Texas, United States of America; 3 Institute of Biology of Komi Science Center of Ural Branch of RAS, Syktyvkar, Russia; 4 Syktyvkar State University, Syktyvkar, Russia; 5 Department of Pathology, University of Washington, Seattle, Washington, United States of America; 6 Moscow Institute of Physics and Technology (State University), Dolgoprudny, Russia; Stanford University Medical Center, United States of America

## Abstract

The common non-steroidal anti-inflammatory drug ibuprofen has been associated with a reduced risk of some age-related pathologies. However, a general pro-longevity role for ibuprofen and its mechanistic basis remains unclear. Here we show that ibuprofen increased the lifespan of *Saccharomyces cerevisiae*, *Caenorhabditis elegans* and *Drosophila melanogaster*, indicative of conserved eukaryotic longevity effects. Studies in yeast indicate that ibuprofen destabilizes the Tat2p permease and inhibits tryptophan uptake. Loss of Tat2p increased replicative lifespan (RLS), but ibuprofen did not increase RLS when Tat2p was stabilized or in an already long-lived strain background impaired for aromatic amino acid uptake. Concomitant with lifespan extension, ibuprofen moderately reduced cell size at birth, leading to a delay in the G1 phase of the cell cycle. Similar changes in cell cycle progression were evident in a large dataset of replicatively long-lived yeast deletion strains. These results point to fundamental cell cycle signatures linked with longevity, implicate aromatic amino acid import in aging and identify a largely safe drug that extends lifespan across different kingdoms of life.

## Author Summary

Aging is the greatest risk factor for many diseases, which together account for the majority of global deaths and healthcare costs. Here we show that the common drug ibuprofen increases the lifespan of yeast, worms and flies, indicative of conserved longevity effects. In budding yeast, an excellent model of cellular longevity mechanisms, ibuprofen's pro-longevity action is independent of its known anti-inflammatory role. We show that the critical function of ibuprofen in longevity is to inhibit the uptake of aromatic amino acids, by destabilizing the high-affinity tryptophan permease. We further show that ibuprofen alters cell cycle progression. Mirroring the effects of ibuprofen, we found that most yeast long-lived mutants were also similarly affected in cell cycle progression. These findings identify a safe drug that extends the lifespan of divergent organisms and reveal fundamental cellular properties associated with longevity.

## Introduction

Levels of cellular and organismal dysfunction increase dramatically with old age. Aging is the greatest risk factor for numerous pathologies, including most forms of cancer, stroke, neurodegenerative disorders, heart disease and diabetes [1]. Hence, delaying aging therapeutically promises immense benefits to human health [2]. However, even with short-lived model organisms, the labor and time associated with unbiased screens for compounds that extend lifespan is a major obstacle [3], [4]. To overcome this drawback, many studies focus on compounds that target pathways already implicated in aging, such as TOR signaling [5], [6], AMP kinase [7], and Sirtuins [8], [9]. Alternatively, phenotypes associated with aging are used as a proxy in screens for potential anti-aging therapeutics [3]. These phenotypes usually include resistance to various types of stress and mitochondrial degeneration, as well as maintenance of proteostasis and genomic stability [10]. The ultimate goal of all these approaches is to identify drugs that will delay the onset of age-related dysfunction and/or provide novel therapeutics to the diseases of aging [2].

Studies of replicative and chronological lifespan in the budding yeast *Saccharomyces cerevisiae* have been driving forces in the identification of conserved genetic pathways that extend lifespan [11], [12]. In this organism, individual cells can be tracked from birth to death [13], with the number of divisions a cell can undergo defining its replicative lifespan (RLS) [14]. The pathways controlling yeast RLS and *C. elegans* lifespan exhibit significant overlap [15]. Hence, aging studies in yeast and other model systems represent invaluable platforms for the discovery of therapeutics that affect aging and a mechanistic dissection of their mode of action.

However, translating leads from aging screens in yeast and other model organisms to drugs that are efficacious and safe in humans represents a significant hurdle [2]. Alternatively, emphasis could be placed on relatively safe compounds that are already used in humans for some indication. One could then ask whether such compounds could extend the lifespan of model organisms. If successful, these drugs would represent excellent candidates for testing in humans for outcomes on healthspan parameters and biomarkers of longevity. They would also serve as invaluable tools to probe conserved longevity pathways, expanding and deepening our understanding of the basic biology of aging.

Here we show that ibuprofen, a common and relatively safe non-steroidal anti-inflammatory drug, extends the lifespan of *S. cerevisiae*, *C. elegans* and *D. melanogaster*. We find that ibuprofen extends the replicative lifespan of yeast cells by destabilizing the high-affinity tryptophan transporter. We also show that ibuprofen causes a small size at birth and a moderate delay in initiation of cell division. Mirroring the effects of ibuprofen, we found that most long-lived yeast mutants were also moderately delayed in initiation of cell division, primarily due to a smaller size at birth. These results point to fundamental cellular properties associated with longevity, and identify a relatively safe drug that alters these properties and extends the lifespan of different species.

## Results

### Ibuprofen extends the lifespan of *S. cerevisiae*, *C. elegans* and *D. melanogaster*

We decided to focus on ibuprofen for three reasons: First, it is a relatively safe over-the-counter medication. Second, ibuprofen may be associated with reduced risk of some age-related pathologies. Third, ibuprofen has not been reported to target any of the known aging pathways (e.g., the TOR or Sirtuin pathways), offering the possibility of novel insights into aging mechanisms. Ibuprofen was invented over 50 years ago. It is the prototypical 2-aryl-propionic acid NSAID. Relative to other NSAIDs, ibuprofen is arguably one of the safest [16]–[19], and is in the World Health Organization's “model list of essential medicines” (18^th^ edition, 2013). As other NSAIDs, ibuprofen has analgesic and anti-pyretic indications. However, these indications stem from ibuprofen's well-established role as a cyclooxygenase inhibitor, interfering with prostaglandin biosynthesis [20]. With regard to age-related pathologies, long-term ibuprofen use reduced the risk of Alzheimer [21] and Parkinson [22], [23] diseases by more than 30%. However, it is unlikely that these beneficial outcomes were solely due to ibuprofen's anti-inflammatory roles because they were not necessarily shared by other NSAIDs. For example, among the NSAIDs examined, ibuprofen showed the most profound reduction in Alzheimer risk (40%), while others, such as celecoxib, had no effect [21]. Similarly, ibuprofen alone, but not other NSAIDs tested, reduced the risk of Parkinson disease [22]. To our knowledge, despite the vast number of studies dealing with ibuprofen, there are no direct measurements of ibuprofen's effects on the lifespan of organisms.

**Figure 1:**
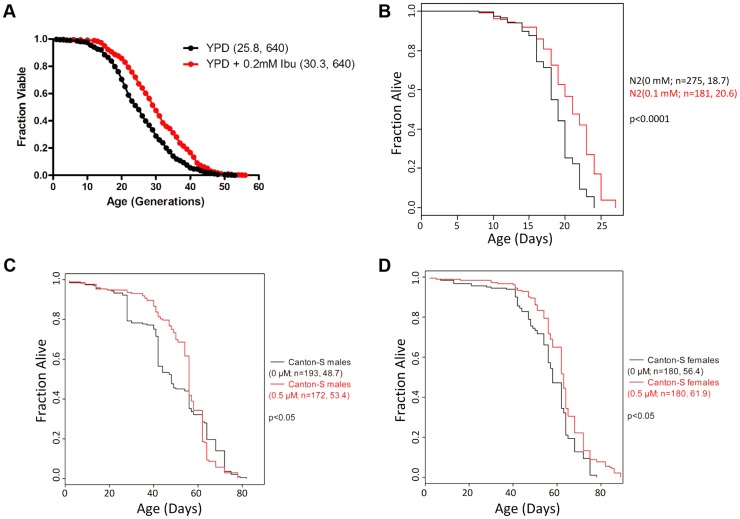
Ibuprofen extends the lifespan of *S. cerevisiae*, *C. elegans* and *D. melanogaster*. **A**, Ibuprofen extends yeast RLS. Survival curves for MATα (BY4742) cells treated with ibuprofen at 0.2 mM (shown in red), compared to experiment-matched untreated cells (shown in black). Mean lifespans are shown in parentheses, along with the number of cells assayed. **B**, Ibuprofen extends the lifespan of *C. elegans*. Survival curves for wild type (N2 strain) animals, treated with ibuprofen at 0.1 mM compared to experiment-matched untreated cells. Mean lifespans are shown in parentheses, along with the number of animals assayed. The data shown are from S1 Table. **C, D,** Ibuprofen extends the lifespan of female *D. melanogaster*. Survival curves for wild type male (**C**) and female (**D**) animals, treated with ibuprofen at 0.05 µM compared to experiment-matched untreated cells. Mean lifespans are shown in parentheses, along with the number of animals assayed. The data shown are from S2 Table.

Consequently, we decided to measure the effects of ibuprofen on yeast replicative lifespan. Added at 0.2 mM, we found that ibuprofen significantly extended the RLS of the standard BY4742 strain background (≈17%, p<0.0001, see Fig. 1A). To test if ibuprofen extends the lifespan of organisms other than yeast, we turned to *C. elegans* for three reasons: First, *C. elegans* is a well-established metazoan aging model, allowing us to gauge the ability of ibuprofen to extend the lifespan of organisms from different kingdoms of life [24]. Second, as in yeast, in *C. elegans* we could probe ibuprofen's effects independently of its role as a cyclooxygenase inhibitor because this organism lacks cyclooxygenase enzymes [25], which are targeted by ibuprofen in mammals [20]. Third, in *C. elegans* ibuprofen has been shown to suppress a phenotype associated with aging, inhibiting the deposition of amyloid β peptide, a marker for Alzheimer disease [26]. We found that animals exposed continuously to varying doses of ibuprofen (0.010–0.4 mM) from hatching until death had a longer lifespan (S1 Table). Note that we used UV-killed bacteria in these experiments, so it is unlikely that these effects are due to indirect effects through the action of ibuprofen on bacterial metabolism (see Materials and Methods). The concentration of ibuprofen at which the lifespan extension was maximal was 0.1 mM (Fig. 1B and S1 Table).

To further test the conservation of the pro-longevity effects of ibuprofen, we asked if the drug could extend the lifespan of *D. melanogaster*, another aging model system. Although the COX gene is absent in *Drosophila*, cyclooxygenase-like activity and inflammatory responses are thought to be present [27]–[29]. We found that ibuprofen (at 0.5 µM) extended the mean and the maximum lifespan of female flies (Fig. 1D and S2 Table). In males, although mean lifespan may also be extended, this was accompanied by a reduction of the maximum lifespan at all doses tested (Fig. 1C and S2 Table). The reasons for the sex-dependent differences in the longevity effects of ibuprofen are not clear. Although the effect in flies is influenced by the sex of the animal, it is nonetheless remarkable that ibuprofen promotes longevity in organisms as divergent as yeast, worms and flies.

Overall, these results suggest that ibuprofen extends lifespan across different kingdoms of life. At least in the case of yeast and worms, the pro-longevity function of ibuprofen is through non-cyclooxygenase-related activity.

### Ibuprofen extends RLS by moderately inhibiting import of aromatic amino acids

**Figure 2:**
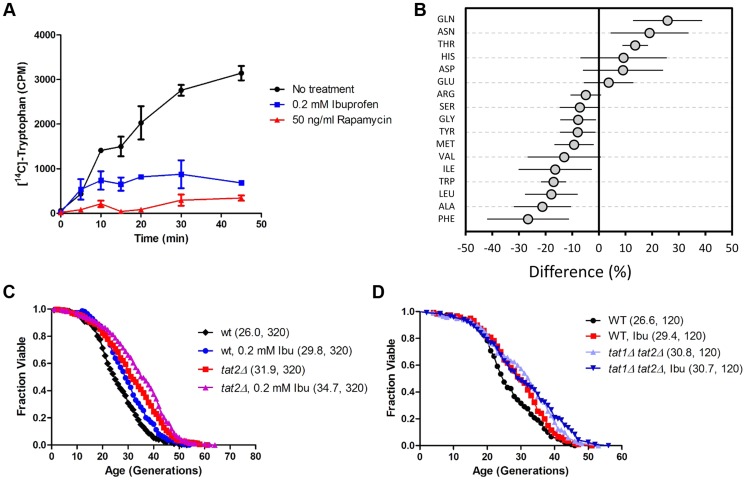
Ibuprofen inhibits tryptophan import, reduces intracellular tryptophan levels and does not extend RLS in the absence of tryptophan permeases. **A**, Uptake of [^14^C]-tryptophan (y axis) is shown at the indicated time points (x axis) after the cells were exposed to, and allowed to internalize, [^14^C]-tryptophan, together with ibuprofen, rapamycin, or no treatment, as indicated. The average of 2 independent experiments is shown, while the error bars indicate the variability between the two experiments. **B**, Intracellular amino acid pools. The percentage difference in the amino acid levels between the ibuprofen-treated (0.2 mM) and untreated (0 mM) samples is shown on the x-axis. The average of six independent experiments (each analyzed in quadruplicate) and the associate standard deviation is shown. All the obtained values from these amino acid analyses are shown in S3 Table. **C**, Survival curves for MATα *tat2Δ* cells (BY4742 strain background) treated with ibuprofen at 0.2 mM (shown in purple), compared to experiment-matched untreated cells (shown in red). For comparison, wild type (WT) cells (BY4742 background) were also included in this experiment and they were either untreated (shown in black) or treated (shown in blue) with ibuprofen. Mean lifespans are shown in parentheses, along with the number of cells assayed. **D**, Survival curves for wild type and *tat1Δ*, *tat2Δ* cells (BY4741 strain background) treated with ibuprofen at 0.2 mM compared to experiment-matched untreated cells. Mean lifespans are shown in parentheses, along with the number of cells assayed.

To understand how ibuprofen might extend lifespan, we focused on the yeast system for the remainder of this report. A previous study interrogated systematically single gene deletions that were sensitive to ibuprofen [30]. Seven out of the eight genes encoding enzymes for de novo synthesis of tryptophan were among the 28 gene deletions that sensitized cells specifically to ibuprofen [30]. To account for these observations, we hypothesized that ibuprofen may interfere with import of aromatic amino acids, including tryptophan. In this scenario, mutants lacking the ability to make tryptophan would rely exclusively on mechanisms responsible for importing tryptophan. Such mutants would be sensitive to any drugs that may impair tryptophan import, perhaps explaining their sensitivity to ibuprofen. To test this model, we then measured directly import of tryptophan in cells treated with ibuprofen. Indeed, ibuprofen inhibited the import of [^14^C]-tryptophan (Fig. 2A). Impaired import of amino acids, including tryptophan, could alter their intracellular pools. Consequently, we measured intracellular levels of amino acids after cells were exposed for 1 hr to 0.2 mM ibuprofen (Fig. 2B), the same dose that extended RLS (see Fig. 1A). We found that the levels of several amino acids were moderately affected by ibuprofen, either increasing, or decreasing, compared to untreated cells (Fig. 2B and S3 Table). Ibuprofen lowered the levels of all aromatic amino acids (Fig. 2B and S3 Table). Overall, these results support the notion that exposing cells to a concentration of ibuprofen that extends RLS inhibits import of aromatic amino acids and lowers their intracellular levels.

Tat2p and Tat1p are the high- and low-affinity tryptophan permeases, respectively [31], [32]. Tat1p is also the high affinity tyrosine permease [31], and may also be involved in the transport of valine and threonine [33]. In addition to tryptophan, Tat2p can transport tyrosine, phenylalanine and, to a lesser extent, alanine and glycine [33]. In agreement with earlier reports [31], tryptophan uptake was moderately inhibited in cells lacking Tat1p, more so in cells lacking Tat2p, and completely blocked in cells lacking both Tat1p and Tat2p (S1 Figure).

To test whether inhibition of aromatic amino acid import is sufficient to extend lifespan, we measured the RLS of *tat1Δ* and *tat2Δ* cells (Fig. 2C). Loss of Tat2p extended RLS significantly (Fig. 2C). We next asked if the ability of ibuprofen to extend RLS depends on aromatic amino acid transport. We found that RLS extension by ibuprofen was attenuated in *tat2Δ* cells and eliminated in cells lacking both Tat1p and Tat2p (Fig. 2D), which cannot import any tryptophan (S1 Figure and [31]). Cyclooxygenase enzymes are not present in yeast [25]. Therefore, ibuprofen must affect yeast cells via unknown off-target mechanisms. Among possible novel mechanisms, our results point to regulation of tryptophan import through Tat2p as a primary conduit by which ibuprofen extends yeast lifespan.

### Ibuprofen extends RLS by destabilizing Tat2p

One of the earliest discovered outputs of the TOR pathway in yeast involves control of tryptophan import and regulation of Tat2p stability [34], [35]. When the Tor1p kinase is active, its downstream effector kinase Npr1p is hyperphosphorylated and inactive. However, inhibiting TOR with rapamycin leads to the dephosphorylation and activation of the Npr1 kinase, which triggers the degradation of Tat2p [36], [37]. Inhibition of TOR activity is a well-characterized, conserved mechanism that delays aging [5], [6]. Furthermore, tryptophan auxotrophs are more sensitive to rapamycin [34] and to ibuprofen [30]. Interestingly, we found that ibuprofen re-sensitized to rapamycin otherwise rapamycin-resistant mutants in the TOR pathway, such as *TOR1-1*, *TOR2-1* and *npr1Δ*, suggesting interactions between ibuprofen and the TOR pathway (S2 Figure). For the above reasons, we examined whether ibuprofen functions through the TOR pathway to inhibit the import of aromatic amino acids and extend RLS.

**Figure 3:**
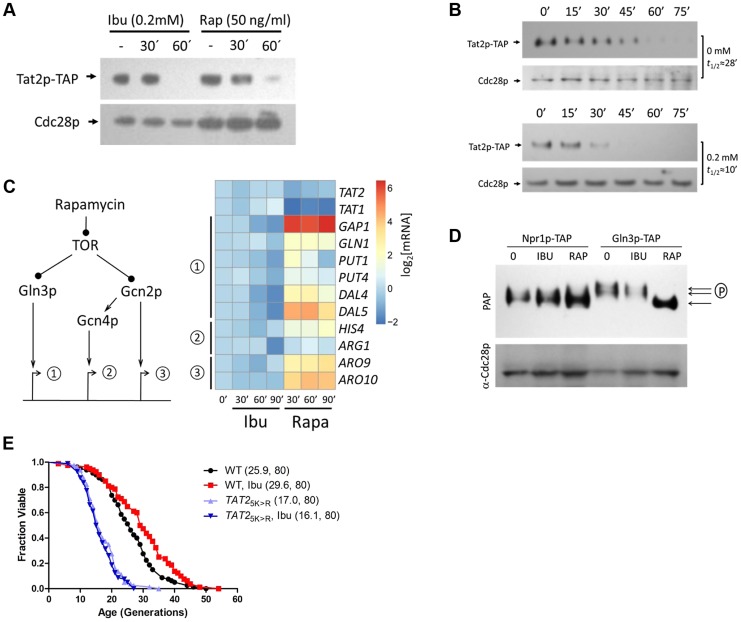
Ibuprofen destabilizes Tat2p without triggering other TOR pathway outputs. **A**, Steady-state Tat2p-TAP levels are reduced upon ibuprofen treatment. Exponentially proliferating cells expressing from its chromosomal location TAP-tagged Tat2p were exposed to ibuprofen or rapamycin at the indicated concentration and for the times shown. Tat2p levels were evaluated by SDS-PAGE and immunoblotting. From the same samples, steady-state levels of untagged Cdc28p are shown for comparison. **B**, Ibuprofen destabilizes Tat2p-TAP. The experiment was performed as in A, except that the cells were treated with cycloheximide at time 0. Tat2p-TAP band densities were quantified with Image J software. These values were then normalized for loading and fitted on an exponential decay function to obtain the half-life values shown on the right of each blot. **C**, Ibuprofen does not trigger gene expression of downstream effectors of the TOR pathway. *Left*, we examined three sets of targets of the TOR pathway, whose expression is known to be affected by rapamycin. *Right*, expression of the targets genes shown was evaluated by qPCR (see Materials and Methods) from exponentially growing cells after addition of ibuprofen (at 0.2 mM) or rapamycin (at 50 ng/ml) at the indicated times. The targets were grouped based on the diagram shown on the left. We also included *TAT1* and *TAT2* expression in this experiment. The average values from at least three experiments in each case were log_2_-transformed and displayed on the heatmap shown using the open-source *pheatmap* package for the R language. **D**, Ibuprofen does not trigger Gln3p dephosphorylation. Cells expressing TAP-tagged variants of two downstream effector of TOR, Npr1p and Gln3p, were treated with ibuprofen or rapamycin for 60 min, at the same concentrations as in A. Npr1p-TAP and Gln3p-TAP levels and mobility, evaluated by immunobloting. **E**, Ibuprofen does not extend the RLS of a strain that carries the stable *2HA-TAT2-5KR* allele. Survival curves of the strains shown treated with ibuprofen at 0.2 mM compared to experiment-matched untreated cells. For comparison, wild type cells carrying a similarly tagged wild type *TAT2* allele (*2HA-TAT2*) were also included in this experiment and they were either untreated (shown in black) or treated (shown in red) with ibuprofen. Mean lifespans are shown in parentheses, along with the number of cells assayed.

Since the TOR pathway controls Tat2p stability and sorting, we examined Tat2p levels in cells treated with ibuprofen. For protein surveillance experiments, we used strains carrying a single allele of the gene of interest, encoding a C-terminal TAP-tagged version of the otherwise wild type ORF, expressed from its native chromosomal location [38]. As reported previously [36], inhibition of TOR by rapamycin reduced Tat2p levels (Fig. 3A). Within 30–60 min after exposure to 0.2 mM ibuprofen, steady-state levels of Tat2p-TAP were reduced to a degree similar to that upon exposure to rapamycin (Fig. 3A). Next, we asked if the reduction in steady-state Tat2p-TAP levels were attributable to destabilization of the protein. We monitored Tat2p-TAP levels after the cells were exposed to cycloheximide, to block new protein synthesis. From these experiments, we estimated that Tat2p-TAP had a half-life of 28 min (Fig. 3B, top panels), consistent with data from a genome-wide study that evaluated with the same approach the stability of TAP-tagged proteins [39]. However, upon addition of 0.2 mM ibuprofen, the half-life of Tat2p-TAP was reduced to about 10 min (Fig. 3B, bottom panels). Next, we measured by qPCR steady-state mRNA levels of *TAT2* and *TAT1* after exposure to ibuprofen or rapamycin. We did not observe a significant difference in the steady-state levels of these mRNAs upon exposure to ibuprofen (Fig. 3C, top). We conclude that in cells treated with ibuprofen the drop in Tat2p levels is likely the result of destabilization of this permease.

Since both rapamycin and ibuprofen destabilize Tat2p, we next asked if ibuprofen affects other TOR-mediated responses. We evaluated known molecular outputs of the TOR pathway after treatment with ibuprofen, in comparison to cells treated with rapamycin. First, we looked at transcriptional outputs (Fig. 3C). Inhibition of TOR by rapamycin is known to trigger expression of mRNAs under the control of the Gln3p and Gcn4p transcription factors [35]. Gcn4p is activated downstream of the Gcn2p kinase. There are also some mRNAs whose transcription is activated in a manner that is Gcn2p-dependent, but Gcn4p-independent [35], [40]. We confirmed all these responses to rapamycin (Fig. 3C). However, exposure to the ibuprofen dose (0.2 mM) that extends RLS did not elicit any of the above TOR-dependent gene expression changes (Fig. 3C). Since rapamycin addition mimics amino acid starvation, triggering increased translation of *GCN4* [41], [42], we also asked if translation of *GCN4* is affected by ibuprofen. For these experiments, we used cells carrying a reporter plasmid with *GCN4* upstream regulatory sequences known to mediate translational control of β-galactosidase expression [43]. Although rapamycin increased β-galactosidase expression significantly, the increase in β-galactosidase expression upon ibuprofen addition was minimal (S3 Figure). These results indicate that ibuprofen at doses that increase RLS does not lead to a general amino acid limitation, consistent with our measurements of intracellular amino acid pools (Fig. 2B and S3 Table).

We also measured different readouts of TOR-dependent signaling in cells treated with ibuprofen at the dose that extends RLS. A downstream effector of TOR is Npr1p, which is dephosphorylated upon rapamycin addition [37], [44]. Whereas Npr1p-TAP appeared to migrate as a single species in rapamycin-treated cells, it migrated as a doublet on SDS-PAGE in both untreated and ibuprofen-treated cells (Fig. 3D). These findings are consistent with TOR-dependent regulation of Npr1p [37], and indicate that ibuprofen likely does not regulate Tat2p stability in an Npr1p-dependent manner. We then examined another TOR effector, the transcription factor Gln3p. Rapamycin triggers the dephosphorylation of Gln3p [35], leading to a fast-migrating species of Gln3p on SDS-PAGE [44], [45]. Such a mobility shift was very pronounced in cells treated with rapamycin but not in cells treated with ibuprofen (Fig. 3D).

Next, we asked if destabilization of Tat2p is required for ibuprofen's ability to extend RLS. Degradation of Tat2p is mediated by ubiquitination at N-terminal sites [36]. *TAT2* alleles encoding Lys→Arg substitutions at five N-terminal Lys residues yield stable Tat2p variants [36]. In contrast to the extended RLS of cells lacking Tat2p (Fig. 2C), cells expressing stabilized Tat2p-5KR from the endogenous chromosomal location had reduced RLS (Fig. 3E). Furthermore, ibuprofen did not extend the RLS of cells expressing stabilized Tat2p-5KR (Fig. 3E). These results suggest that destabilization of Tat2p is required for lifespan extension by ibuprofen.

**Figure 4:**
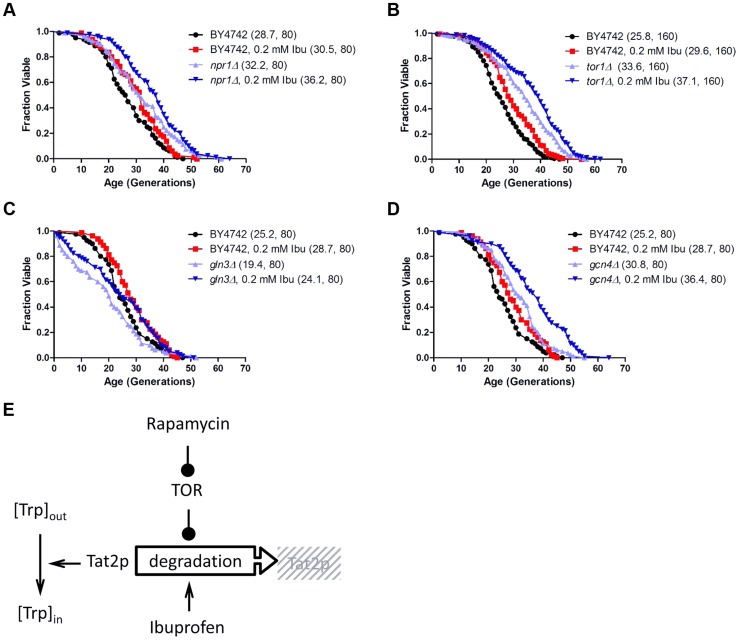
Ibuprofen extends RLS of TOR pathway mutants. Ibuprofen extends the RLS of *npr1Δ* (**A**), *tor1Δ* (**B**), *gln3Δ* (**C**) and *gcn4Δ* (**D**) cells. Survival curves of the strains shown (BY4742 background) treated with ibuprofen at 0.2 mM compared to experiment-matched untreated cells. Mean lifespans are shown in parentheses, along with the number of cells assayed. **E**, Model of the mechanism of function of ibuprofen, acting antagonistically but at least in part independently of the TOR pathway to destabilize Tat2p and inhibit tryptophan import in the cells.

All the results described above indicate that although ibuprofen destabilizes Tat2p, a known target of TOR, it does so without significantly altering additional outputs of the TOR pathway. Consequently, we next tested if ibuprofen extends RLS in the context of TOR pathway mutants. Even though *tor1Δ* mutants are long-lived [46], their RLS were increased further by ibuprofen (Fig. 4). Ibuprofen also extended the RLS of *npr1Δ, gln3Δ and gcn4Δ* cells, demonstrating that the corresponding gene products are not required for its longevity-promoting effects (Fig. 4). Taken together, these results suggest that the mechanism of RLS extension we described for ibuprofen converges on the stability of the Tat2p permease, which is also targeted by the TOR pathway (Fig. 4E).

### Ibuprofen treatment reduces cell size at birth and delays the G1 phase of the cell cycle

**Figure 5:**
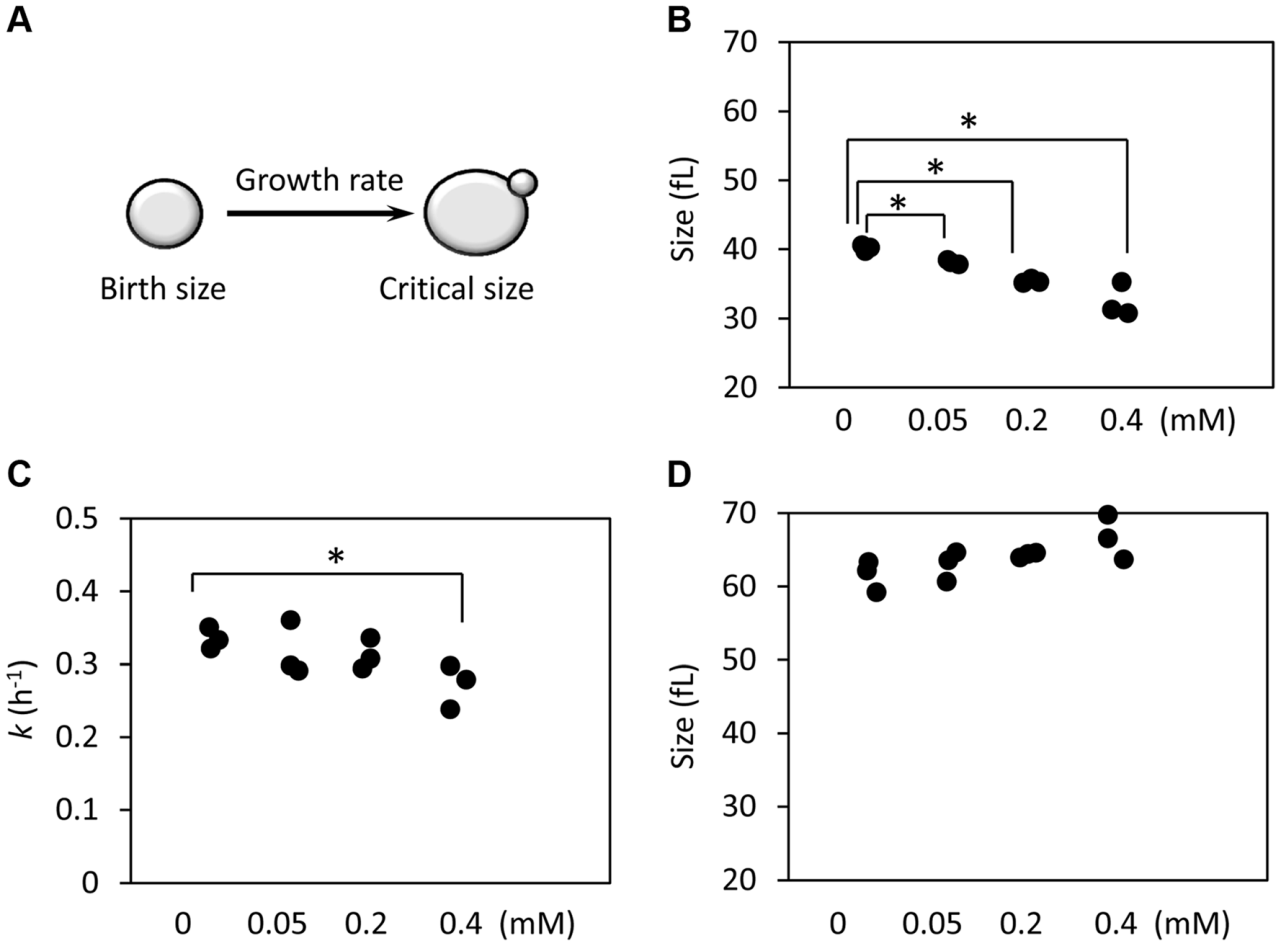
Ibuprofen at low doses moderately delays G1, primarily through a reduction in birth size. **A**, Schematic of the variables that determine the length of the G1 phase. **B**, The birth size of BY4743 cells exposed at different doses of ibuprofen was measured from three independent experiments in each case, similar to the ones shown in S4 Figure. Asterisks indicate statistically significant differences compared to the untreated samples (p<0.05, from Student's *t* tests). **C**, The specific rate of cell size increase constant *k* (in h^−1^) was measured from the elutriation experiments shown in S5 Figure, assuming exponential growth. **D**, The critical cell size of the indicated strains (shown in fl), was measured from the same elutriation experiments shown in C and S5 Figure.

Since preservation of a proliferative state is at the core of cellular replicative lifespan, we decided to examine if exposure to ibuprofen alters cell cycle kinetics. We used centrifugal elutriation to obtain highly synchronous, unbudded, early G1 cell populations. We scored these cultures over time microscopically and with a channelyzer, a particle counter that directly measures the volume of cells. Note that in yeast initiation of DNA replication is coupled to the formation of a bud [47]. Thus, one can monitor the timing of initiation of division by phase microscopy. We calculated the specific rate of size increase (which we call here “growth rate”) and critical size (the size at which half of the cells budded). We then incorporated measurements of birth size, as we described previously [48], [49]. Knowing how small the cells are when they are born, how big they have to get before they can divide, and how fast they grow from their birth size to their critical size, determines the absolute length of the G1 phase (Fig. 5A). With this methodology, we examined in synchronous cultures G1 progression upon treatment with varying doses of ibuprofen (Fig. 5B and S5 Figure). At lower doses, ibuprofen caused a dose-dependent delay in G1 progression, mostly due to reduction in birth size (Fig. 5 and S4 Figure). Ibuprofen also significantly compromised growth rate at 0.4 mM or higher (Fig. 5C and S5 Figure). At even higher doses, ibuprofen was toxic to yeast cells in this medium (not shown). These results suggest that at the dose that ibuprofen extends RLS, it also reduces cell size at birth and moderately delays the G1 phase of the cell cycle.

### Correlation of cell cycle parameters with RLS

We next asked whether the cell cycle alterations caused by ibuprofen reflect more general links between cell cycle progression and RLS. To answer this question, we first queried systematic genome-wide datasets that report on these processes. Soon after systematic panels of yeast deletion mutants were generated, with each strain lacking a nonessential gene, they were assayed for their competitive fitness [50], and for the mean cell size of asynchronously dividing cell populations [51]. Recently, we also measured by flow cytometry the DNA content of these mutants [48] and calculated the birth size of the newborn daughter cells [49]. Together these studies provide phenotypes that are associated with cell cycle progression (i.e., fitness, mean cell size, DNA content, and birth size) for 3,979 single-gene deletion mutants. We have also initiated a systematic measurement of the RLS of all these mutants [46], but this effort is still ongoing. Nonetheless, in the Saccharomyces Genome Database (http://www.yeastgenome.org/), 137 deletion mutants in the same background have already been studied and classified as having increased RLS, based on published data from our group and others (see S1 Dataset, sheet “variables”, for a list of these ORFs and the corresponding variables). Collectively, these datasets permit a much broader evaluation of possible links between cell cycle progression and RLS.

**Figure 6:**
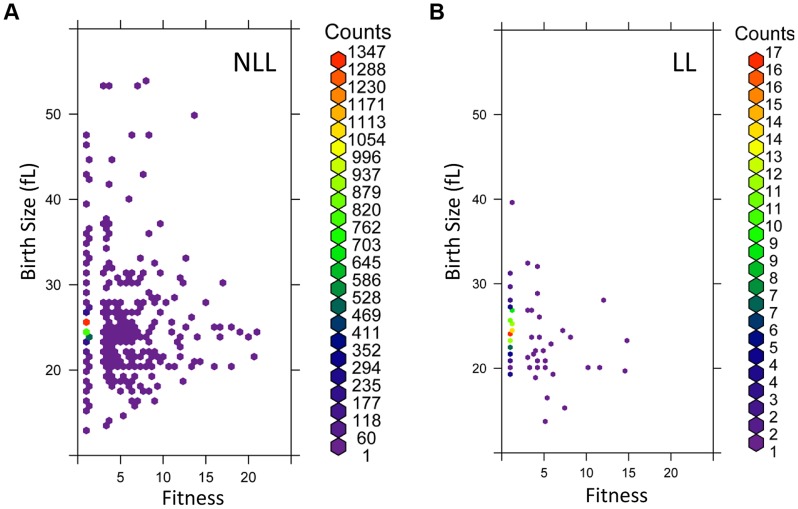
LL mutant birth size and fitness are more constrained than those of NLL mutants. **A**, The birth size values (y axis) from all 3,842 NLL mutants from S1 Dataset were plotted against their corresponding fitness values (x axis). Because many data points were overlapping, they were binned using the hexbin function of the R software package. The displayed colors represent the number of strains within each bin, as indicated by the color key to the right. **B**, The birth size values (y axis) from all 137 LL mutants from S1 Dataset were plotted against their corresponding fitness values (x axis), as in A. The number of strains in each bin (counts) are shown on the right.

We compared the cell cycle related phenotypes of the 137 long-lived (LL) mutants with those of the remaining 3,842 not long-lived (NLL) strains (S1 Dataset). We found that there was not a highly significant difference in the mean cell size of the two groups (S6 Figure and S1 Dataset). However, there were small but significant differences in the birth size of newborn cells (S1 Dataset, based on both the parametric Student's t test and the non-parametric Mann-Whitney test). Overall, it appears that LL mutants have a smaller birth size and reduced fitness, which likely accounts for the moderate increase in the relative duration of the G1 phase (S1 Dataset and S6 Figure). To better visualize these differences, we plotted in a density scatter plot the birth size values against the corresponding fitness for each mutant, for the LL and NLL groups (Fig. 6). From these data, we conclude that although LL mutants have a slightly smaller birth size and reduced fitness, these relationships are constrained and not proportional. Compared to NLL mutants, LL mutants were neither the smallest, nor the least fit. To evaluate the extent that each of the above cell cycle parameters could serve as predictors for long RLS, we also performed binary logistic regression analysis (see S1 Dataset, sheet “Statistics”). In the context of all the variables we analyzed, the best predictor for long RLS was daughter birth size (p = 0.000613), followed by fitness (p = 0.026).

**Figure 7:**
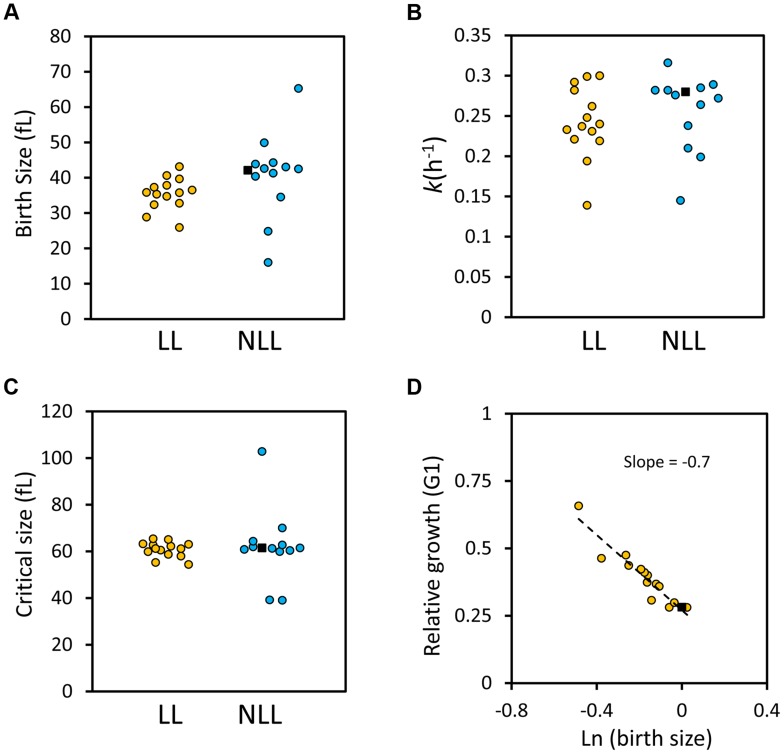
G1 phase cell cycle parameters of LL and NLL strains. **A**, The birth size values (y axis) of the 14 LL and 13 NLL strains shown in S4 Table. The filled black squares correspond to the values of the wild type control. The data from S4 Table were used to generate the graphs shown. **B**, The specific rate of cell size increase (*k*, shown on the y axis) for the LL and NLL strains shown in S4 Table was calculated from synchronous, elutriated cultures (see Materials and Methods). **C**, The critical size (y axis) of the LL and NLL strains shown in S4 Table was calculated from the same experiments shown in B. **D**, LL mutants have efficient cell size control mechanisms. On the x axis is the logarithm of the normalized birth size values of the LL mutants shown in S4 Table, plotted against their relative growth in size during the G1 phase of each strain (*k*T_G1_, y axis). The dashed line is a linear fit obtained with the regression function of Microsoft Excel. The filled square is the wild type strain.

The phenotypes we examined above were from cells dividing asynchronously. Next, we evaluated synchronous cell populations, to measure parameters that determine the absolute length of the G1 phase of the cell cycle and the timing of initiation of a new round of cell division. For this analysis, we compared a group of 14 LL strains to 13 NLL strains (see S4 Table). The mutants we chose lack ORFs that function in diverse cellular processes, including metabolism, protein synthesis, growth signaling or transcription (see S4 Table). From these experiments with synchronous cultures, we conclude the following: Most LL mutants had a smaller birth size (p = 0.026, based on a Mann-Whitney test), but they appear to occupy a “sweet-spot” since strains with much reduced birth size were not long-lived (Fig. 7A and S4 Table). However, at least for the LL mutants we examined here, the size at which they initiated division was normal (Fig. 7C and S4 Table). Many LL mutants also had a reduced growth rate (Fig. 7B and S4 Table) but this difference was not statistically significant (Mann-Whitney test). Overall, these observations support our conclusion from the genome wide datasets of parameters from asynchronous cultures that smaller daughter birth size is a phenotype associated with increased RLS (Fig. 6 and S1 Dataset). Furthermore, the fact that critical size is not significantly altered in the long-lived mutants we evaluated probably explains why the mean cell size of asynchronous cultures is not a good predictor of long RLS.

Next, we asked about the efficiency of size control mechanisms in the LL mutants. Plotting the logarithm of birth size against the relative growth in the G1 phase of the cell cycle is a measure of the efficiency of cell size control mechanisms [52], [53]. In such plots, a slope of zero indicates no size control. From single-cell analysis, wild type budding yeast daughter cells display a negative slope of 0.7 [52], [53]. We applied this methodology to all the synchronous daughter cell populations of the LL strains shown in S4 Table and also obtained a slope of −0.7 (Fig. 7D). We conclude that the LL mutants we analyzed displayed cell size control that appeared to be as efficient as that of the wild type strain (Fig. 7D). In the Discussion, we comment on the implications of all these data, in the context of recent models of cellular aging.

**Figure 8:**
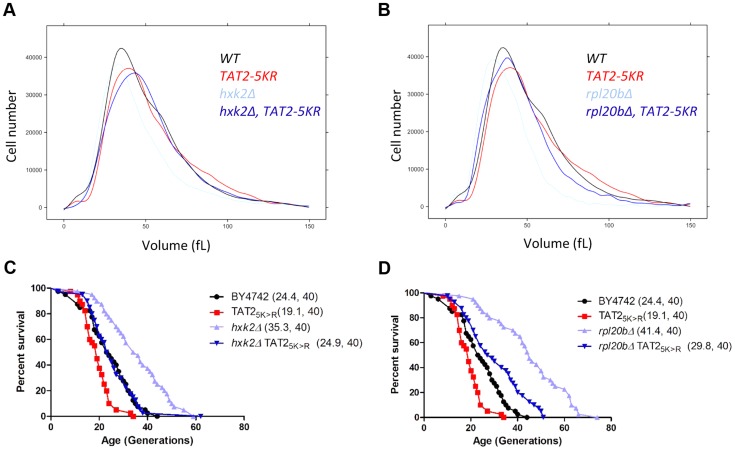
Stabilization of Tat2p increases birth size and suppresses replicative lifespan extension in *hxk2Δ* and *rpl20bΔ* cells. **A** and **B**, The cell size of the indicated haploid cell populations cultured in YPD (2% Dextrose) medium was measured using a channelyzer. Cell numbers are plotted on the y axis and the x axis indicates size (in fL). **C** and **D**, Experiment-matched survival curves of the same strains shown in A and B. Mean lifespans are shown in parentheses, along with the number of cells assayed.

To follow our other findings, we also interrogated possible connections among tryptophan levels, birth size, Tat2p stability and replicative lifespan. We found that although the effects of exogenous tryptophan on cell size were minimal (S7A Figure), exogenous tryptophan suppressed ibuprofen's pro-longevity effects (S7B Figure). Furthermore, steady-state levels of epitope-tagged Tat2p-TAP were not reduced in the *hxk2Δ* and *rpl20bΔ* mutants with small birth size and increased RLS, and appeared instead to be increased by ≈2 fold (S8 Figure). We also noted that there was no disproportionate decrease in tryptophan levels in *hxk2Δ* and *sch9Δ mutants* (S5 Table), which have a small birth size, and they are long-lived (S4 Table). Instead, these mutants have lower levels (≈20%–60%) of nearly all amino acids, including tryptophan (S5 Table), resembling growth-limited cells in that regard [54]. However, stabilization of Tat2p increased birth size (Fig. 8A, B) and suppressed the long lifespan of mutants with small birth size (Fig. 8C, D). Taken together, these results suggest that a link between small birth size and tryptophan levels is not straightforward. Replicative longevity in yeast is not always accompanied with a small birth size, lower Tat2p abundance and lower tryptophan levels. Nonetheless, stabilization of Tat2p both increases the size of cells at birth, and attenuates the pro-longevity effects upon loss of Hxk2p or Rpl20Bp.

Lastly, we asked whether interfering with the expression of aromatic amino acid transporters could increase the lifespan of animals. To this end, we tested whether using RNAi to suppress expression of either one of two putative orthologs of Tat2p could extend the lifespan of *C. elegans*, resembling the long lifespan of yeast *tat2Δ* mutants. These worm orthologs are encoded by genes C50D2.2 and F23F1.6, and the corresponding gene products are 24%, and 20%, identical to Tat2p, respectively. We found that suppressing expression of C50D2.2 significantly increased both the mean and the maximal lifespan of the RNAi-treated animals (mean lifespan increased ≈15%, p<0.0001), compared to the control animals (S9A Figure). Animals treated with RNAi against F23F1.6 also lived longer, albeit the effect was less pronounced (mean lifespan increased ≈5%, p = 0.0006, see S9B Figure). These results suggest that targeting amino acid transport mechanisms may have general pro-longevity effects.

## Discussion

We will first consider the implications of our findings connecting ibuprofen with tryptophan import and lifespan. We will also discuss our results in the context of previous models linking cell cycle progression with RLS.

### Ibuprofen: A safe NSAID as a longevity therapeutic?

Although ibuprofen had not been tested for its effects on lifespan, other common NSAIDs have been examined. Aspirin slightly extended the lifespan of genetically heterogeneous male mice [54]. In the same study, a nitrosylated flurbiprofen analog had no effect on the lifespan of mice [54]. Even against cyclooxygenases, NSAIDs often display different modes of inhibition and specificity against specific isoforms. For example, flurbiprofen causes irreversible inhibition of cyclooxygenase activity while ibuprofen does not [20]. Another NSAID that has been reported to extend the lifespan of *C. elegans* is celecoxib [55]. Similar to our results with ibuprofen, celecoxib extended lifespan when added from hatching until death [55]. We also noted that the effective pro-longevity concentrations of ibuprofen were much lower in flies than in worms or yeast (0.5 µM vs. 100–200 µM, respectively; see Fig. 1). The reason for this difference is unclear at present. In healthy humans who took a 600 mg ibuprofen dose up to four times daily, the peak plasma concentration was around 50 µg/ml, corresponding to 240 µM [56]. In another study, a single 400 mg dose of ibuprofen results in a plasma concentration of 8.4 µg/ml, or 40 µM [57]. Therefore, the levels of ibuprofen that extend the lifespan of worms and yeast are in the range of ibuprofen levels reached in people taking the drug at typical doses. Overall, our results add to the growing role of NSAIDs, and ibuprofen in particular. These compounds are relatively safe therapeutics that may combat age-related pathologies and extend the lifespan of divergent organisms, from yeast to invertebrates and possibly mammals.

### Aromatic amino acid uptake: At the nexus of aging mechanisms?

We discovered that ibuprofen inhibits tryptophan import, cells lacking the high affinity tryptophan transporter are long-lived, and ibuprofen's longevity effects depend on its ability to destabilize the high affinity tryptophan transporter (Figs. 2, 3). How important is tryptophan uptake in meeting the needs of cells for this amino acid? Metazoans cannot make tryptophan and rely exclusively on tryptophan uptake. Plants and microbes, including yeast, can synthesize tryptophan through the shikimate and chorismate pathways [58]. However, tryptophan is by far the costliest amino acid to synthesize, consuming 78 mol of ATP for 1 mol of tryptophan [58]. Hence, given a choice, it is likely that tryptophan uptake will be preferable to synthesis, even in tryptophan prototrophs. Consistent with a role for tryptophan uptake in aging, low tryptophan diets extend the lifespan of rodents [59]. Perhaps in line with our finding that worms with suppressed expression of the yeast tryptophan permease orthologs live longer (S9 Figure), tryptophan analogs that may inhibit the tryptophan transport system have also been reported to increase the lifespan of flies [60]. Conversely, an increase in degradation of tryptophan through the kynuverine pathway has been associated with accelerated aging in animals (reviewed in [61]). Tryptophan's roles are not limited to protein synthesis. For example, in animals, kynuverine metabolites serve as immune and neuronal modulators [61], [62] and affect cell viability in tissue culture [63]. However, it is not clear if tryptophan degradation and its involvement in the above pathways are a cause or consequence of aging.

Our results expand the role of aromatic amino acids in aging. We demonstrate that interfering with aromatic amino acid uptake, genetically or pharmacologically, can extend lifespan. Furthermore, to our knowledge, the extended lifespan of *tat2Δ* cells (Fig. 2) is the first report of an amino-acid permease deletion that extends RLS in yeast. Perhaps this is a reflection of the incomplete systematic evaluation of the RLS of permease mutants. Alternatively, it may be due to the low abundance of tryptophan in the cells, coupled to the energetic cost of synthesizing tryptophan. Tryptophan levels are 5–10 times lower than the levels of the next low-abundance amino acids (tyrosine and methionine, see S3 Table). Finally, we would like to note that when ibuprofen was added at the dose that extended RLS, the drop in tryptophan levels was 15–20% (Fig. 2B and S3 Table). Hence, the cells were not severely limited for tryptophan, or any other amino acid (Fig. 2B and S3 Table), consistent with the lack of significant *GCN4* de-repression (S3 Figure). As we comment later, perhaps this is yet another manifestation of hormesis, underpinning ibuprofen's effects in RLS.

Our results strongly suggest not only that ibuprofen destabilizes the Tat2p permease (Fig. 3), but also that this is the critical function of ibuprofen in mediating RLS extension since stabilization of Tat2p neutralized the ability of ibuprofen to extend lifespan (Fig. 3E). Stabilization of Tat2p also suppressed replicative lifespan extension by loss of Hxk2p or Rpl20Bp (Fig. 8). The TOR pathway is known to control the stability of Tat2p. A few years after the discovery of the TOR genes in yeast [64], it was reported that increased levels of *TAT2* conferred resistance to the macrolide FK506 [31], [34]. Then, TOR activity was shown to inhibit turnover of Tat2p, through Npr1p, a downstream effector kinase of TOR [36], [37]. Interestingly, however, loss of Npr1p does not stabilize Tat2p, presumably because in the absence of Npr1p other kinases substitute for Npr1p's role [37]. In any case, in light of all this information, it seemed plausible that ibuprofen increased RLS through the TOR pathway. Indeed, ibuprofen conferred rapamycin sensitivity to *TOR* gain-of-function mutants or cells lacking Npr1p (S2 Figure). Such strains are normally resistant to rapamycin and have increased Tat2p activity. We noted however that at the dose that extended RLS, ibuprofen did not elicit molecular responses consistent with TOR pathway inhibition (Fig. 3 and S3 Figure). Furthermore, ibuprofen extended the RLS of both *tor1Δ* and *npr1Δ* strains (Fig. 4), suggesting that it acts through a mechanism that is at least partially distinct from reduced TOR signaling. It is possible that ibuprofen, through its destabilization of Tat2p and inhibition of aromatic amino acid import, may further sensitize the TOR pathway and exacerbate the longevity effects of TOR pathway inhibition. Alternatively, ibuprofen may be acting in parallel with the TOR pathway, to destabilize Tat2p, inhibit import of aromatic amino acids, and extend RLS (Fig. 4E).

### Cell cycle parameters and RLS: Hypertrophy or antagonistic pleiotropy and hormesis?

As we detail next, our results argue against the hypertrophy model of cellular aging. Before initiating a new round of cell division, newborn daughter cells reach a critical size threshold. Critical size is characteristic of the yeast strain and medium used. Yeast mother cells also increase in size with every successive division [65], [66]. The hypertrophy model of aging proposed that mother cells reach their replicative potential once they attain a fixed maximal size, beyond which they cannot divide any more [67], [68]. This terminal and very large cell size may represent the point at which cells have become so large that they cannot sustain functions necessary for cell division, perhaps due to a very low surface to volume ratio or for other reasons.

The hypertrophy model makes a clear prediction: For small cells, it would take extra divisions to reach the terminal size, resulting in longer RLS. Conversely, large cells will reach the terminal size after fewer divisions, having a shorter RLS. Furthermore, changes in RLS and cell size ought to be proportional. Specifically, it was proposed that RLS is simply a quotient of the difference between maximal and critical size values, and the increase in cell size per cell division [67]. The hypertrophy model received a significant boost from a recent report of changes in RLS that were indeed strongly proportional to observed changes in size [69]. For example, a plot of cell diameter at birth against RLS displayed a linear fit with a coefficient of determination (R^2^) equal to 0.96 [69]. However, it was pointed out that the above conclusions supporting a role for hypertrophy in RLS were drawn from a small sample of mutants (<15), of which no more than a handful were long-lived [70], [71]. Short RLS can be due to many causes, with a significant portion unlinked to the mechanistic events driving RLS [70], [72]. We based our conclusions on genome-wide datasets, parsed in two groups: mutants with a longer lifespan against all the rest (S1 Dataset). We found that the mean cell size of the two groups was similar (S1 Dataset and S6 Figure). This finding argues against a key prediction of the hypertrophy model: LL mutants would have a small overall cell size, which would in turn enable these cells to divide more times until they reach the terminal size.

Yang et al also reported that the birth size of long-lived cells was significantly and proportionally smaller than that of short-lived cells [69]. Here, we also identified significant differences in birth size between LL and NLL mutants, but these differences were slight and they were not linearly proportional (S1 Dataset, S4 Table and Figs. 6,7). There are at least two reasons that may account for these discrepancies: First, the sample sizes were vastly different. Second, birth size was measured with different methodologies. Yang et al used photomicroscopy after micromanipulation to measure cell diameters and then extrapolate to calculate the birth size of those cells [69]. Instead, we relied on channelyzer measurements, which report directly on volume, to obtain the size of the smallest cells in dividing populations [49].

Longer RLS has been associated with reduced fitness [73] and slower growth rate [69]. We also noticed that LL mutants have reduced fitness (S1 Dataset and Fig. 6) and growth rate (Fig. 7 and S4 Table). However, these differences were again modest and constrained. The majority of the smallest and/or slowest growing mutants were not long-lived (Fig. 6, and as an example see *sfp1Δ* cells, S4 Table). We would also like to note that none of the 14 LL mutants we analyzed with detailed synchronous cell cycle profiles had significantly altered critical size (Fig. 7 and S4 Table). Based on our data, it seems that LL mutants are born smaller and/or grow slightly slower, but they reach a normal critical size before initiating division. This is consistent with the observation that LL and NLL mutants have similar mean size (S1 Dataset and S6 Figure). In fact, their size control appears intact and indistinguishable from wild type cells (Fig. 7D). Again, these observations do not support the hypertrophy model.

The cell cycle patterns we described above argue that in most cases long lifespan is associated with a moderate delay in cell cycle progression early in life. This delay arises from a small birth size and/or slower growth rate. As a result of these changes, LL mutants have a G1 delay (S4 Table and S6 Figure). As was noted previously, these observations are perhaps in line with the antagonistic pleiotropy model [73]. In that scenario, mutations that increase lifespan have opposite effects at different ages. A small delay in cell cycle progression would have adverse effects in young cells, decreasing their rates of division, but be beneficial in older cells, enabling them to divide more times. It is also possible that our results can be explained from the viewpoint of hormesis. In hormetic situations, a treatment at low intensity or dose can be beneficial, but at higher levels, the same treatment is harmful. Hormetic responses to various types of stress are often associated with lifespan extension [74]. Perhaps a moderate delay in G1 progression produces such beneficial effects in lifespan. At the same time, it is not difficult to see why a more severe delay would be detrimental. Hormetic considerations may explain why the cell cycle differences we observed in LL mutants are moderate and constrained, but not severe and proportional. This is illustrated by the effects of ibuprofen: At low doses, ibuprofen causes a moderate cell cycle delay, mimicking the profile of LL mutants (Fig. 5), and extends lifespan (Fig. 1A). At higher doses, however, ibuprofen delays cell proliferation more severely (Fig. 5). Regardless of the models invoked, our data suggest that using cell cycle parameters may be a promising and readily scalable approach to identify interventions that extend RLS.

In conclusion, the results we report reveal unexpected cellular properties associated with longevity and demonstrate that novel functions of existing safe therapeutics can extend the longevity of organisms from different kingdoms of life.

## Methods

### Strains and media

The strains we used are shown in S6 Table. Single gene deletion mutants in the BY4741, BY4742, or BY4743 strains were generated by the Yeast Deletion Project [50]. Double mutants (e.g., strain CHY01) were constructed from crosses of the corresponding single mutants, each in the background of opposing mating types (BY4741 and BY4742), followed by tetrad dissection, growth on selective media lacking lysine and methionine, and genotyping by PCR.

The *2HA-TAT2* and *2HA-TAT2-5KR* plasmids pAS55 and pTB355, respectively, were gifts from Dr. Michael Hall [36]. To generate strains carrying these alleles integrated in the chromosome, we performed the following: First, with one-step PCR replacement [75], in strain BY4742 we replaced the *TAT2* allele with *URA3*, yielding a *tat2Δ::URA* strain (CHY02). Then, we PCR-amplified the *TAT2* ORF and flanking sequences from plasmids pAS55 and pTB355, using primers that correspond to upstream (Forward primer: 5′- CCTTCTGAGTGACGCTTAAACCATCTGCAAGTCTCTTCCGCGGTGATGACGGTGAAAACC-3′) and downstream (Reverse primer: 5′- GACGCGAATTGTTTCACACGGTAGGATAAGAGAAATTGCGGACGTTGTAAAACGACGGCC-3′) flanking sequences. These PCR products were then used to transform strain CHY02, counter-selecting for the loss of the *URA3* marker on plates containing 5-Fluoroorotic Acid (5-FOA). The resulting transformants, CHY03 and CHY04, were genotyped by PCR to confirm the presence of the *2HA-TAT2* and *2HA-TAT2-5KR* alleles, respectively, expressed from the endogenous *TAT2* chromosomal location. Note that the epitope tag did not alter the lifespan-related function of Tat2p, because the *2HA-TAT2* strain had the same RLS as the untagged but otherwise identical strain and its lifespan was further extended by ibuprofen (Fig. 3E). With one-step PCR replacement [75], in strain CHY04 (carrying the *2HA-TAT2-5KR* allele), we replaced the *HXK2*, or *RPL20B*, ORFs with *URA3*, yielding strains CHY05, or CHY06, respectively. Similarly, using strain 202233243 (carrying the *TAT2-TAP* allele) we generated strains CHY07, CHY08, CHY09, lacking *HXK2*, *RPL20B*, or *SCH9*, respectively.

Unless indicated otherwise, the medium we used in most experiments, including cell cycle and RLS measurements, was YPD (1% ^w^/_v_ yeast extract, 2% ^w^/_v_ peptone, 2% ^w^/_v_ dextrose). We used the ibuprofen sodium salt (Sigma, Cat#: I1892) dissolved in water to a 0.1 M stock solution, from which it was added to autoclaved media as indicated in each case. Rapamycin (Sigma, Cat#: R0395) was dissolved in ethanol to a 1 mg/ml stock, before it was added to autoclaved media as indicated.

### Cell cycle and RLS measurements

We have described in detail elsewhere the methodology for elutriation experiments [48], birth size measurements [49] and RLS assays [13]. The smoothened cell size histograms we show are the splines of the corresponding raw data, generated with the R software package “lattice”. All RLS experiments were carried out on standard YPD plates.

### Tryptophan uptake assays

Cells from an early logarithmic culture in YPD medium were divided in three. Drugs were added as indicated and the cultures were incubated at 30°C for another 3 hrs. The three culture fractions were treated as indicated (mock, 0.2 mM ibuprofen or 50 ng/ml rapamycin), and they were incubated for an additional 3 h at 30°C. Cultures were harvested by centrifugation and washed twice in 10 mM sodium citrate, pH 5.5. The cell pellets were resuspended in ice-cold uptake buffer (10 mM sodium citrate (pH 5.5); 20 mM (NH4)_2_SO_4_; 2% glucose). The cell densities were measured and normalized to 5×10^7^ cells/ml. While samples were on ice, they were treated again as before (mock, ibuprofen at 0.2 mM, or rapamycin at 50 ng/ml). We then added radiolabelled tryptophan solution (L-Tryptophan, [side chain-3-^14^C], 40–60 mCi/mmol, Moravek Biochemicals, Cat#: MC402). The cell cultures were then incubated at 30°C for the times shown. At the indicated time points, 0.1 ml aliquots were transferred to microcentrifuge tubes containing 1 ml ice-cold uptake solution to stop the uptake. The samples were centrifuged for 10 sec and the cell pellets were washed three times (centrifuging for 10 sec in between) with ice-cold uptake buffer. Finally, the cell pellets were resuspended in 0.1 ml of uptake buffer and transferred to a vial containing 5 ml of scintillation mixture. The retained radioactivity was then quantified by liquid scintillation using a Beckman LS6500 Multipurpose Scintillation Counter.

### Amino acid analysis

Cells were grown in rich YPD medium until they reached a density of 1–5×10^6^ cells/ml. For drug treatment, the culture was then divided in half and one part was treated with ibuprofen at 0.2 mM for 1 hr. After quenching with sodium azide (at 0.1%) and cycloheximide (at 50 µg/ml), the cells were collected by centrifugation and washed with water (1∶20 volume, compared to the original culture volume). The cells were collected again by a brief spin (10 sec in a microfuge), and resuspended in 1∶100 volume of water (compared to the original culture volume). Then, the cell suspension was boiled for 5 min and centrifuged to collect the supernatant, representing the metabolite extract. This extract was then analyzed by standard PTH-derivatization and HPLC analysis [76] at the Texas A&M University Protein Chemistry Facility, to quantify the nmoles of each amino acid present in the extract. These values were normalized for the starting cell density and reported in S3 Table.

### Immunoblotting

Protein extracts for immunoblots were made with the NaOH extraction method [77]. The extracts were run on 4–12% gradient SDS-PAGE gels. For detection of proteins of interest on immunoblots we used a rabbit polyclonal anti-Cdc28 antibody (SantaCruz, Cat#: sc28550) at a 1∶500 dilution to detect Cdc28p, a mouse monoclonal anti-PSTAIR antibody (Abcam, Cat#: ab10345) at a 1∶1000 dilution to detect Cdk, and the peroxidase-anti-peroxidase (PAP) soluble complex (Sigma, Cat#: P1291) at a 1∶1000 dilution to detect TAP-tagged proteins. Conjugated anti-rabbit and anti-mouse secondary antibodies and chemiluminescence reagents were from Thermo Scientific, and used at the dilutions recommended by the manufacturer.

### Quantitative real-time PCR analysis

Cells were grown to early logarithmic phase in YPD medium and treated with 0.2 mM ibuprofen or 50 ng/ml rapamycin for 0 min, 30 min, 60 min and 90 min, as indicated. Cells were harvested and cell extracts were prepared using the glass bead lysis method [75]. Total RNA was purified using the QIAGEN RNeasy kit (Cat#: 74106) according to the manufacturer's instructions. 1 mg RNA was reverse-transcribed using Bio-Rad's iScript cDNA synthesis kit (Cat#: 170-8890) containing oligo (dT) and random hexamer primers. Lysate preparation, RNA purification and reverse transcription were performed on multiple biological samples in parallel. cDNA products were amplified with a Roche LightCycler 480 using SYBRGreen I Master (Roche, Cat#: 04887352001) for detection according to manufacturer's recommendations. Primer sequences are listed in S7 Table. cDNA of 0 min treatment was used as standard for normalization.

### β-galactosidase assays

The p180 reporter plasmid driving expression of β-galactosidase was a gift from Dr. Alan Hinnebusch [43]. The plasmid was transformed into BY4741 cells, and transformants were selected on plates lacking uracil. Overnight cultures were diluted 1∶100 in synthetic complete medium lacking uracil. After incubation for 3 h at 30°C, cell cultures were treated as indicated (mock, 0.2 mM ibuprofen or 50 ng/ml rapamycin) and incubated for an additional 3-4 h at 30°C. We examined three independent cultures for each treatment. Cells were harvested and cell extracts were prepared in Z buffer (60 mM Na_2_HPO_4_.7H_2_O, 40 mM NaH_2_PO_4_.H_2_O, 10 mM KCl, 1 mM MgSO_4_.7H_2_O, 50 mM β-mercaptoethanol) with glass bead lysis. Protein concentrations were measured using Bradford assay. Cell extracts were diluted in Z buffer after normalizing for the same amount of protein. We then added 0.1 ml of cell extract into 96-well plates. To each well, we then added 20 µl of ο-nitrophenyl-β-D-galactopyranoside (ONPG, Sigma Cat#: 73660) solution (4 mg/ml in Z buffer). Plates were incubated at 30°C for 40–60 min until the color of the samples became pale yellow. OD_450 nm_ for each sample was measured using a SpectraMax 190 Absorbance Microplate Reader.

### *C. elegans* lifespans

All the experiments were done as described elsewhere [78]. From the single-cell egg stage, lifespans were monitored on plates seeded with UV-killed bacteria (strain OP-50). Other than scoring times, during which the animals were moved to room temperature, at all stages of these experiments the animals were kept at 20°C. RNA interference (RNAi) was delivered to worms as described previously [79]. RNAi feeding bacteria were kind gifts from Dr. Gordon Lithgow. Control animals were fed bacteria carrying an empty vector (strain pAD12). Lifespan experiments were performed in the second generation of animals grown on RNAi bacteria.

### *D. melanogaster* lifespans

Wild-type strain Canton-S were obtained from the Bloomington *Drosophila* Stock Center (Indiana University, Bloomington, Indiana, USA) and used in lifespan experiments. Flies were kept under standard conditions, at 25°C, in a 12∶12 hour light-dark regime, on an agar/semolina/sugar/yeast medium [80]. 25 pairs of parents with synchronized 24 h egg laying, were used to obtain the experimental flies. The flies were extracted from vials immediately after imago eclosion. 150–200 flies were collected (approximately 30 adult flies per 50 ml vial) for each experimental variant. Males and non-virgin females were kept separately. Flies were put in vials with medium contained ibuprofen (Sigma Cat#: I110) at concentration of 0.3, 0.5 and 1 µM, and transferred to a fresh medium with ibuprofen twice weekly. Dead flies were scored daily during *Drosophila* lifetime.

The data was used to plot survival curves and to calculate the mean, median, minimum and maximum lifespan and the age of 90% mortality calculated with the open source R software package. In order to estimate the significant statistical differences between experimental and control groups, log-rank tests were used. The significance of differences in maximum lifespan was evaluated using the Wang-Allison test. Following the Wang-Allison test, each animal in each experiment was categorized into one of two groups: either lifespan above the 90th percentile or lifespan below the 90th percentile. A two by two contingency table was used to record data. An ordinary χ^2^-test was used for independent testing of two groups [81].

## Supporting Information

S1 Figure Tryptophan uptake is inhibited in cells lacking Tat1p and Tat2p. Uptake of [^14^C]-tryptophan (y axis) is shown at the indicated time points (x axis) after the indicated strains were exposed to, and allowed to internalize, [^14^C]-tryptophan.
(JPG)

S2 Figure Cells relying on tryptophan uptake are sensitive to ibuprofen. Strains that are tryptophan auxotrophs (*trp1Δ* in the BY4743 background and all strains in the Jk9-3d background, see S6 Table) were sensitive to growth on solid medium containing ibuprofen (compare the 1^st^ and 2^nd^ plates). Strains carrying dominant gain-of-function *TOR1* or *TOR2* alleles, or lacking Npr1p are resistant to rapamycin (see [37] and this figure *top*, compare the 1^st^ and 4^th^ plates). Interestingly, ibuprofen re-sensitized these strains to rapamycin (*bottom*, 2^nd^ plate). Therefore, when cells rely on tryptophan uptake (e.g., in Trp auxotrophs, or when TOR is inhibited, which through Npr1p affects Tat2p) they are sensitive to ibuprofen. Adding additional exogenous tryptophan suppressed partially, albeit not completely, these effects of ibuprofen. All strains were spotted at 5-fold serial dilutions on YPD plates with the indicated chemicals, from the same starting cell densities. The plates were incubated at 30°C, for 3 days and they were photographed at the same time. Ibuprofen was added at 0.2 mM; Trp at 100 µg/ml; and Rapamycin at 50 ng/ml.
(JPG)

S3 Figure Ibuprofen does not relieve translational repression of *GCN4* expression. Absorbance associated with β-galactosidase expression (y axis) for wild type cells carrying the p180 lacZ reporter plasmid and treated as indicated (x axis).
(JPG)

S4 Figure Ibuprofen reduces the mean and birth cell size. The cell size of exponentially proliferating cell populations (BY4743), cultured in YPD (2% Dextrose) medium, and treated with the indicated dose of ibuprofen was measured using a channelyzer. Cell numbers are plotted on the y axis and the x axis indicates size (in fL).
(JPG)

S5 Figure Determining the length of G1 in cells treated with ibuprofen. **A**, Graphs from which we determined the specific rate of cell size increase constant *k*, shown in Fig. 5C. We plotted the natural log of the cells size (y axis), against time (shown in hours, x axis). Measurements were from synchronous BY4743 cultures, in rich (YPD-2% Dextrose) medium. After each elutriation, the elutriated culture was split in four fractions. Ibuprofen was then added to each fraction, to the final concentration shown in each case. **B**, Graphs of the fraction of budded cells (y axis) as a function of cell size (in fl, x axis), from the same elutriation experiments. The data points used to determine the critical size for division we show in Fig. 5D were from the linear portion of each experiment, when the percentage of budded cells began to increase.
(JPG)

S6 Figure Kernel density plots of mean size and G1 DNA content of LL vs. NLL mutants. **A**, The G1 DNA content in LL vs. NLL mutants was plotted as a density plot using the open source R software package, to better visualize the distribution of this variable in the two groups of mutants. **B**, The mean cell size of LL vs. NLL mutants was plotted as a density plot using the open source R software package, as in A.
(JPG)

S7 Figure Exogenous tryptophan does not affect cell size, but it suppresses replicative lifespan extension by ibuprofen. **A**, The cell size of haploid cell populations (BY4741 background) cultured in YPD (2% Dextrose) medium at the indicated tryptophan concentrations was measured using a channelyzer. Cell numbers are plotted on the y axis and the x axis indicates size (in fL). **B**, The replicative lifespan extension (in percentage) of cells treated with 0.2 mM ibuprofen compared to their untreated counterparts (BY4742 background) is shown at the indicated concentrations of Trp, Phe or Tyr added in the medium (YPD, with 2% Dextrose). All the data were from experiment-matched survival curves from at least 40 cells assayed in each case.
(JPG)

S8 Figure Steady-state Tat2p-TAP levels in mutants with small birth size. Tat2p levels in exponentially proliferating cells expressing from its chromosomal location TAP-tagged Tat2p and lacking the indicated genes were evaluated by SDS-PAGE and immunoblotting. From the same samples, steady-state levels of untagged Cdk, detected with an anti-PSTAIR antibody, are shown for comparison. Overall loading is also shown from the Ponceau-stained blot.
(JPG)

S9 Figure Interfering with the expression of putative amino acid transporters extends the lifespan of *C. elegans*. Survival curves for animals fed with bacteria carrying an RNAi vector against C50D2.2 (**A**) or F23F1.6 (**B**) compared to control animals fed with bacteria carrying the empty vector. Mean lifespans are shown in parentheses, along with the number of animals assayed. The p value shown associated with each experiment was calculated as in S1 Table.
(JPG)

S1 Dataset Genome-wide dataset of cell cycle parameters in long-lived (LL) and not-long-lived (NLL) deletion strains lacking non-essential genes (spreadsheet “Variables”) and the associated statistical correlations (spreadsheet “Statistics”). The LL ORFs were listed in SGD at the time of preparing this manuscript that when deleted in the S288c strain background increased RLS. All the non-essential deletion mutants we included in this analysis were from the dataset in [49]. Among these, there were 137 deletion mutants in the S288c strain background classified as long-lived in replicative lifespan assays (see S1 Table). The remaining 3,842 mutants of the dataset in [49] were not long-lived (NLL).
(XLSX)

S1 Table Summary of *C. elegans* lifespans.
(DOCX)

S2 Table Summary of *D. melanogaster* lifespans.
(DOCX)

S3 Table Intracellular amino acid levels upon ibuprofen treatment.
(DOCX)

S4 Table Cell cycle parameters from synchronous cultures of LL and NLL homozygous diploid deletion strains.
(DOCX)

S5 Table Intracellular amino acid levels in *hxk2Δ* and *sch9Δ* cells.
(DOCX)

S6 Table *S. cerevisiae* strains used in this study.
(DOCX)

S7 Table Primer pairs for quantitative real-time PCR.
(DOCX)
