# Supplementary figures and images for: Enhanced Longevity by Ibuprofen, Conserved in Multiple Species, Occurs in Yeast through Inhibition of Tryptophan Import
Source: PLoS Genet. 2014 Dec 18;10(12):e1004860. doi: 10.1371/journal.pgen.1004860 (PMC4270464; doi:10.1371/journal.pgen.1004860)

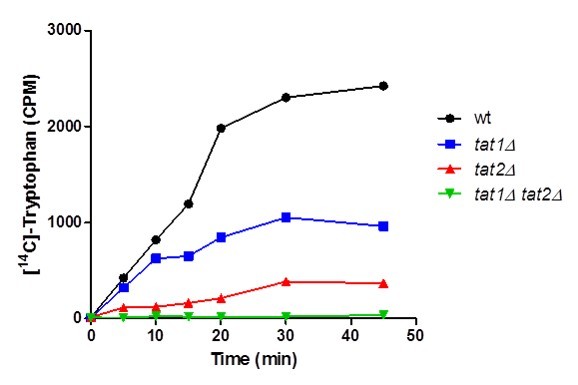

Supplement: S1 Figure — Tryptophan uptake is inhibited in cells lacking Tat1p and Tat2p. Uptake of [14C]-tryptophan (y axis) is shown at the indicated time points (x axis) after the indicated strains were exposed to, and allowed to internalize, [14C]-tryptophan. (JPG) [file pgen.1004860.s001.jpg]

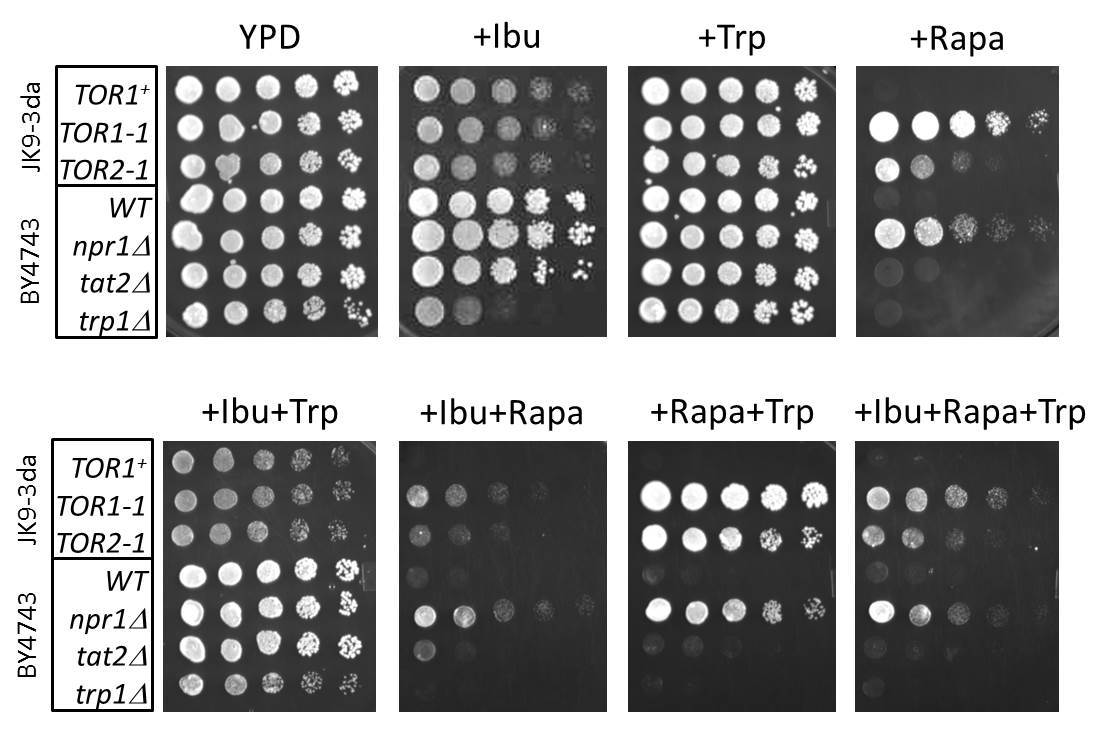

Supplement: S2 Figure — Cells relying on tryptophan uptake are sensitive to ibuprofen. Strains that are tryptophan auxotrophs (trp1Δ in the BY4743 background and all strains in the Jk9-3d background, see S6 Table) were sensitive to growth on solid medium containing ibuprofen (compare the 1st and 2nd plates). Strains carrying dominant gain-of-function TOR1 or TOR2 alleles, or lacking Npr1p are resistant to rapamycin (see [37] and this figure top, compare the 1st and 4th plates). Interestingly, ibuprofen re-sensitized these strains to rapamycin (bottom, 2nd plate). Therefore, when cells rely on tryptophan uptake (e.g., in Trp auxotrophs, or when TOR is inhibited, which through Npr1p affects Tat2p) they are sensitive to ibuprofen. Adding additional exogenous tryptophan suppressed partially, albeit not completely, these effects of ibuprofen. All strains were spotted at 5-fold serial dilutions on YPD plates with the indicated chemicals, from the same starting cell densities. The plates were incubated at 30°C, for 3 days and they were photographed at the same time. Ibuprofen was added at 0.2 mM; Trp at 100 µg/ml; and Rapamycin at 50 ng/ml. (JPG) [file pgen.1004860.s002.jpg]

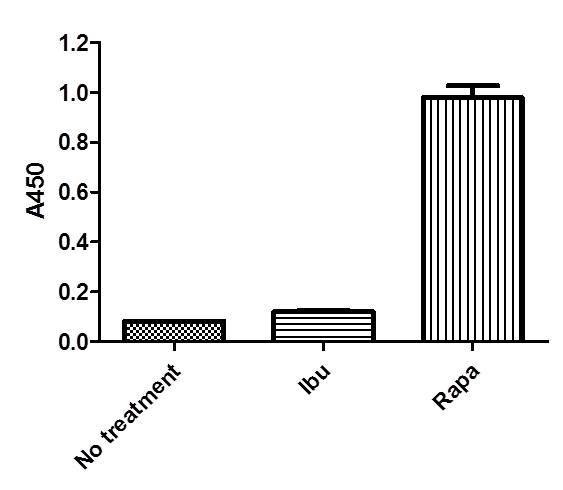

Supplement: S3 Figure — Ibuprofen does not relieve translational repression of GCN4 expression. Absorbance associated with β-galactosidase expression (y axis) for wild type cells carrying the p180 lacZ reporter plasmid and treated as indicated (x axis). (JPG) [file pgen.1004860.s003.jpg]

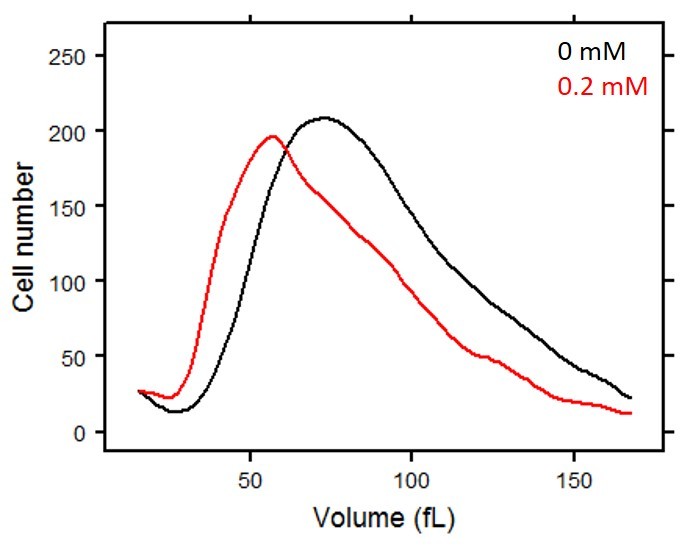

Supplement: S4 Figure — Ibuprofen reduces the mean and birth cell size. The cell size of exponentially proliferating cell populations (BY4743), cultured in YPD (2% Dextrose) medium, and treated with the indicated dose of ibuprofen was measured using a channelyzer. Cell numbers are plotted on the y axis and the x axis indicates size (in fL). (JPG) [file pgen.1004860.s004.jpg]

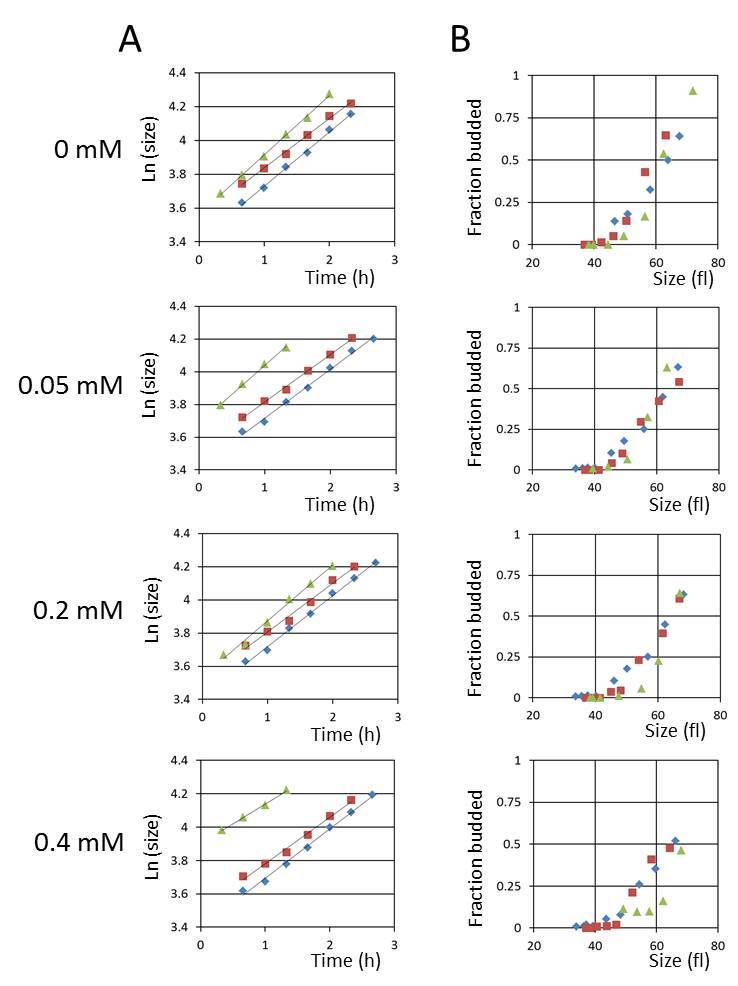

Supplement: S5 Figure — Determining the length of G1 in cells treated with ibuprofen. A, Graphs from which we determined the specific rate of cell size increase constant k, shown in Fig. 5C. We plotted the natural log of the cells size (y axis), against time (shown in hours, x axis). Measurements were from synchronous BY4743 cultures, in rich (YPD-2% Dextrose) medium. After each elutriation, the elutriated culture was split in four fractions. Ibuprofen was then added to each fraction, to the final concentration shown in each case. B, Graphs of the fraction of budded cells (y axis) as a function of cell size (in fl, x axis), from the same elutriation experiments. The data points used to determine the critical size for division we show in Fig. 5D were from the linear portion of each experiment, when the percentage of budded cells began to increase. (JPG) [file pgen.1004860.s005.jpg]

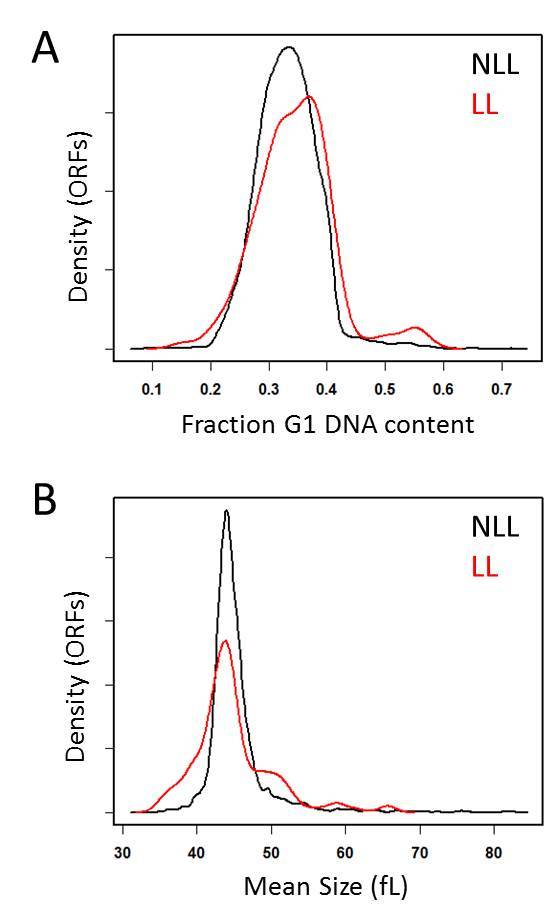

Supplement: S6 Figure — Kernel density plots of mean size and G1 DNA content of LL vs. NLL mutants. A, The G1 DNA content in LL vs. NLL mutants was plotted as a density plot using the open source R software package, to better visualize the distribution of this variable in the two groups of mutants. B, The mean cell size of LL vs. NLL mutants was plotted as a density plot using the open source R software package, as in A. (JPG) [file pgen.1004860.s006.jpg]

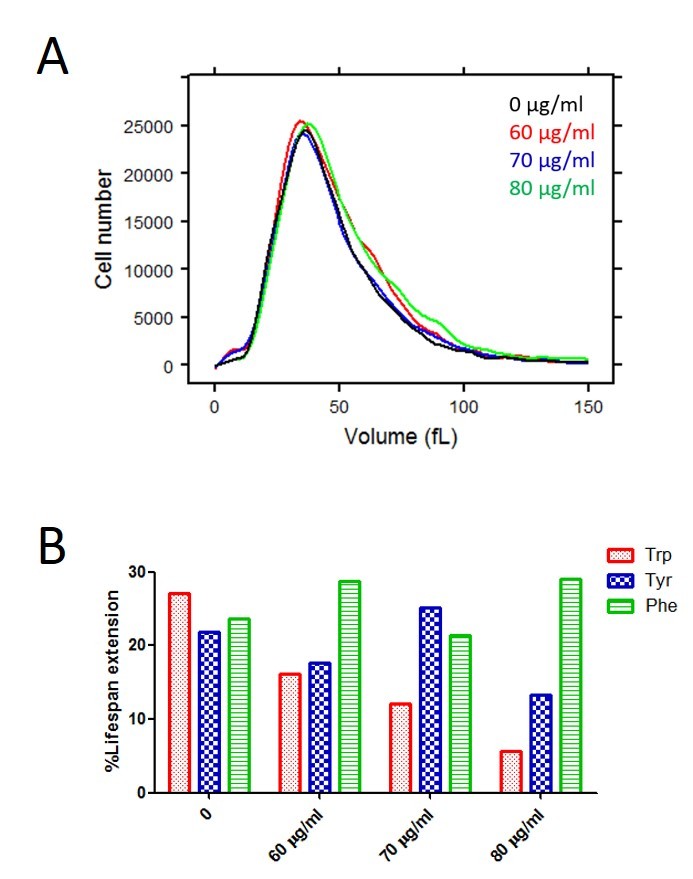

Supplement: S7 Figure — Exogenous tryptophan does not affect cell size, but it suppresses replicative lifespan extension by ibuprofen. A, The cell size of haploid cell populations (BY4741 background) cultured in YPD (2% Dextrose) medium at the indicated tryptophan concentrations was measured using a channelyzer. Cell numbers are plotted on the y axis and the x axis indicates size (in fL). B, The replicative lifespan extension (in percentage) of cells treated with 0.2 mM ibuprofen compared to their untreated counterparts (BY4742 background) is shown at the indicated concentrations of Trp, Phe or Tyr added in the medium (YPD, with 2% Dextrose). All the data were from experiment-matched survival curves from at least 40 cells assayed in each case. (JPG) [file pgen.1004860.s007.jpg]

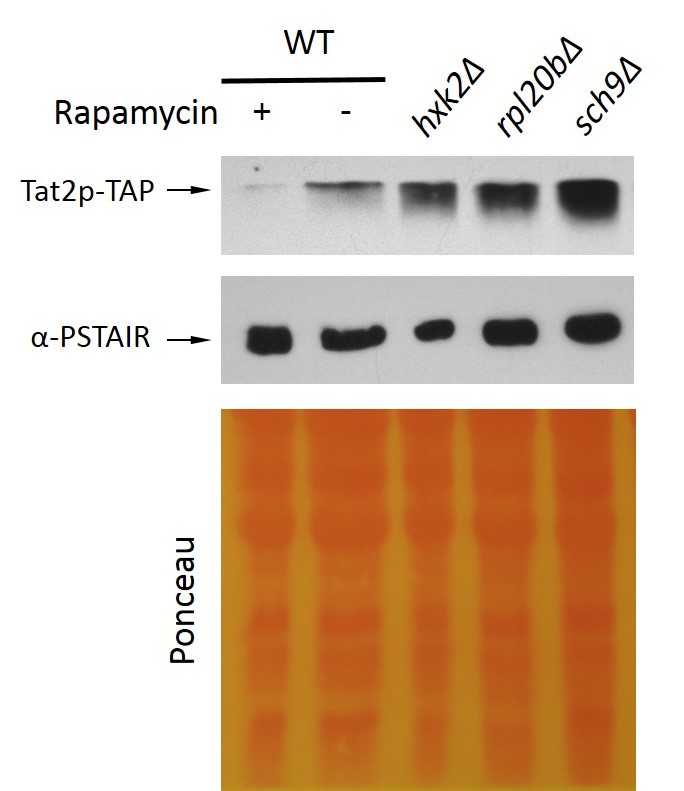

Supplement: S8 Figure — Steady-state Tat2p-TAP levels in mutants with small birth size. Tat2p levels in exponentially proliferating cells expressing from its chromosomal location TAP-tagged Tat2p and lacking the indicated genes were evaluated by SDS-PAGE and immunoblotting. From the same samples, steady-state levels of untagged Cdk, detected with an anti-PSTAIR antibody, are shown for comparison. Overall loading is also shown from the Ponceau-stained blot. (JPG) [file pgen.1004860.s008.jpg]

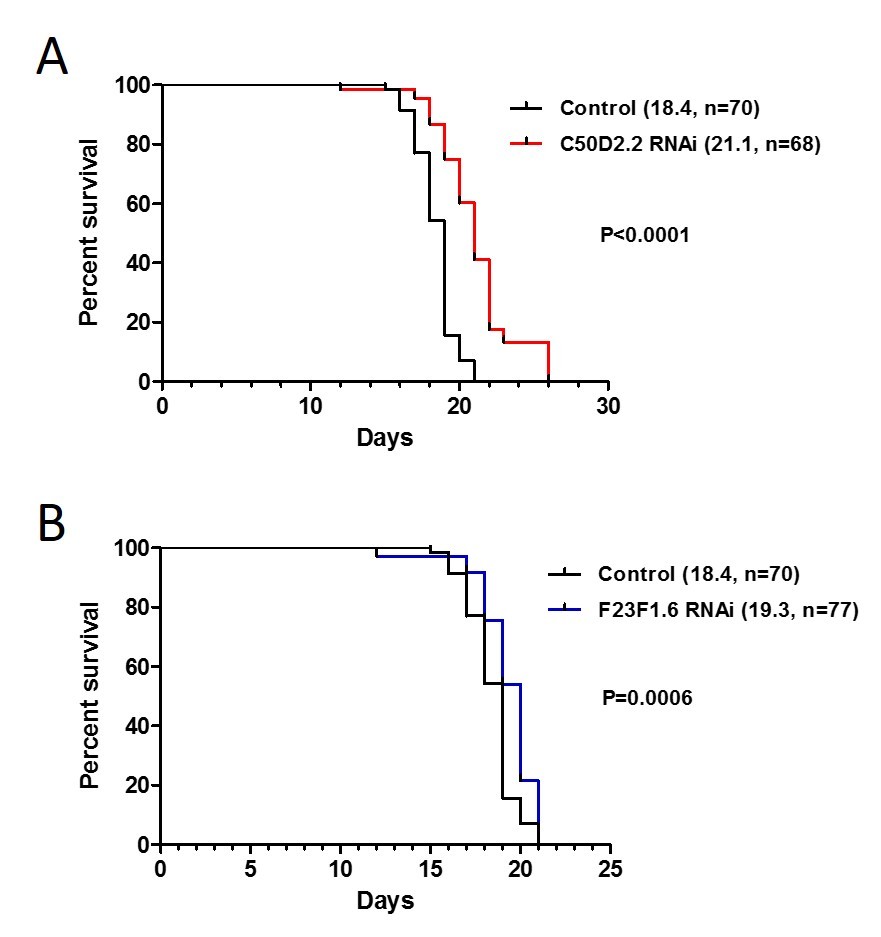

Supplement: S9 Figure — Interfering with the expression of putative amino acid transporters extends the lifespan of C. elegans. Survival curves for animals fed with bacteria carrying an RNAi vector against C50D2.2 (A) or F23F1.6 (B) compared to control animals fed with bacteria carrying the empty vector. Mean lifespans are shown in parentheses, along with the number of animals assayed. The p value shown associated with each experiment was calculated as in S1 Table. (JPG) [file pgen.1004860.s009.jpg]
