# Supplementary material for: Enhanced Longevity by Ibuprofen, Conserved in Multiple Species, Occurs in Yeast through Inhibition of Tryptophan Import
Source: PLoS Genet. 2014 Dec 18;10(12):e1004860. doi: 10.1371/journal.pgen.1004860 (PMC4270464; doi:10.1371/journal.pgen.1004860)
Supplement: S1 Table — Summary of C. elegans lifespans. (DOCX) [file pgen.1004860.s011.docx]

**Table S1. Summary of *C. elegans* lifespans**

| **Strain** | **Ibuprofen (mM)** | **N** | **Mean**^a^ | **Median**^a^ |
| --- | --- | --- | --- | --- |
| N2 | 0 | 275 | 18.656 | 19 |
| N2 | 0.010 ^b^ | 107 | **19.52** | 19 |
| N2 | 0.025 ^b^ | 107 | **20.461** | 21 |
| N2 | 0.050 ^b^ | 113 | **19.988** | 20 |
| N2 | 0.100 ^b^ | 181 | **20.617** | 21 |
| N2 | 0.200 ^b^ | 107 | 20.036 | 21 |
| N2 | 0.400 ^b^ | 106 | **19.956** | 20 |
| N2 (adults) | 0.100 ^c^ | 85 | 18.625 | 20 |

^a^The mean and median values shown were calculated with the survival functions of the open source R software package, using the Kaplan-Meier estimator. Values shown in bold were significantly different (p<0.05) from matched controls, based on log-rank tests calculated with the open source R software package.

^b^In these experiments ibuprofen was added in the plates from the egg laying stage, and maintained for the remainder of the experiment.

^c^In these experiments adult 2 day old animals were exposed to ibuprofen added in the plates, and maintained for the remainder of the experiment.
