# Supplementary material for: Enhanced Longevity by Ibuprofen, Conserved in Multiple Species, Occurs in Yeast through Inhibition of Tryptophan Import
Source: PLoS Genet. 2014 Dec 18;10(12):e1004860. doi: 10.1371/journal.pgen.1004860 (PMC4270464; doi:10.1371/journal.pgen.1004860)
Supplement: S2 Table — Summary of D. melanogaster lifespans. (DOCX) [file pgen.1004860.s012.docx]

**Table S2. Summary of *D. melanogaster* lifespans**

| **Sex** | **Ibuprofen (μM)** | **N** | **Mean**^a^ | **Median**^a^ | **90 %** ^b^ |
| --- | --- | --- | --- | --- | --- |
| ♂♂ | 0 | 193 | 48.7 | 48 | 72 |
| ♂♂ | 0.3 | 192 | 49.2 | 48 | **62** |
| ♂♂ | 0.5 | 172 | 53.4 | 56 | **64** |
| ♂♂ | 1.0 | 182 | 50.5 | 51 | **64** |
| ♀♀ | 0 | 180 | 56.4 | 58 | 72 |
| ♀♀ | 0.3 | 191 | 58.7 | 62 | **78** |
| ♀♀ | 0.5 | 180 | **61.9^c^** | 63 | **75** |
| ♀♀ | 1.0 | 182 | 56.5 | 58 | **68** |

^a^The mean and median values shown were calculated from the corresponding survival functions.

^b^The values of the age of 90% mortality are shown as maximum lifespan parameter. The significance of differences in maximum lifespan was evaluated using the Wang-Allison test. Values shown in bold were significantly different (p<0.05) from matched controls.

^c^The mean lifespan shown was significantly different (p<0.05) from matched controls, based on log-rank, Kolmogorov-Smirnov and Gehan-Breslow-Wilcoxon tests.
