# Supplementary material for: Enhanced Longevity by Ibuprofen, Conserved in Multiple Species, Occurs in Yeast through Inhibition of Tryptophan Import
Source: PLoS Genet. 2014 Dec 18;10(12):e1004860. doi: 10.1371/journal.pgen.1004860 (PMC4270464; doi:10.1371/journal.pgen.1004860)
Supplement: S3 Table — Intracellular amino acid levels upon ibuprofen treatment. (DOCX) [file pgen.1004860.s013.docx]

**Table S3. Intracellular amino acid levels upon ibuprofen treatment**

| **AA** | **Untreated (fmoles/cell)^a^** | **Ibuprofen-treated (fmoles/cell)^b^** | **Relative ratio^c^** | ***t* test (p)^d^** |
| --- | --- | --- | --- | --- |
| Asp | 0.4690±0.2520 | 0.5111±0.3179 | 1.09 | 0.273781 |
| Glu | 2.4596±1.4166 | 2.5496±1.4902 | 1.04 | 0.259022 |
| Asn | 0.0904±0.0412 | 0.1076±0.0644 | 1.19 | 0.162928 |
| Ser | 0.5334±0.2902 | 0.4953±0.2628 | 0.93 | 0.050921 |
| Gln | 0.3104±0.1888 | 0.3904±0.2827 | 1.26 | 0.108496 |
| His | 0.2317±0.0927 | 0.2531±0.0968 | 1.09 | 0.208756 |
| Gly | 0.4675±0.2118 | 0.4311±0.1854 | 0.92 | 0.115199 |
| Thr | 0.4433±0.2844 | 0.5037±0.3105 | 1.14 | 0.003631 |
| Ala | 0.426±0.2072 | 0.3409±0.1729 | 0.79 | 0.017098 |
| Arg | 1.2210±0.5524 | 1.1611±0.5471 | 0.95 | 0.055155 |
| Tyr | 0.0367±0.0189 | 0.0338±0.0173 | 0.92 | 0.037445 |
| Val | 0.2120±0.1148 | 0.1844±0.1099 | 0.87 | 0.047499 |
| Met | 0.0558±0.0277 | 0.0506±0.0253 | 0.91 | 0.040671 |
| Trp | 0.0080±0.0050 | 0.0067±0.0038 | 0.83 | 0.043891 |
| Phe | 0.1303±0.0746 | 0.0956±0.0568 | 0.73 | 0.052613 |
| Ile | 0.1568±0.0886 | 0.1311±0.0798 | 0.84 | 0.058517 |
| Leu | 0.2271±0.1240 | 0.1866±0.1033 | 0.82 | 0.022676 |

^a,b^Cells were cultured and harvested as described in Materials and Methods.

^c^The relative ratio of ibuprofen-treated vs. untreated is shown.

^d^Student’s *t* tests between untreated and treated samples were paired, 2-tailed, assuming unequal variance, and they were calculated with the corresponding function in Microsoft Excel. The p value associated with each test is shown.
