# Supplementary material for: Enhanced Longevity by Ibuprofen, Conserved in Multiple Species, Occurs in Yeast through Inhibition of Tryptophan Import
Source: PLoS Genet. 2014 Dec 18;10(12):e1004860. doi: 10.1371/journal.pgen.1004860 (PMC4270464; doi:10.1371/journal.pgen.1004860)
Supplement: S4 Table — Cell cycle parameters from synchronous cultures of LL and NLL homozygous diploid deletion strains. (DOCX) [file pgen.1004860.s014.docx]

**Table S4. Cell cycle parameters from synchronous cultures of LL and NLL homozygous diploid deletion strains^a^.**

| [**Strain**](http://polywiki.tamu.edu/wiki/bin/view/PolymenisLab/AgingElutriationExperiments?sortcol=0;table=1;up=0#sorted_table) | [**Birth**](http://polywiki.tamu.edu/wiki/bin/view/PolymenisLab/AgingElutriationExperiments?sortcol=4;table=1;up=0#sorted_table)  [**size (fL)**](http://polywiki.tamu.edu/wiki/bin/view/PolymenisLab/AgingElutriationExperiments?sortcol=4;table=1;up=0#sorted_table) | ***k*** [**(specific rate of**](http://polywiki.tamu.edu/wiki/bin/view/PolymenisLab/AgingElutriationExperiments?sortcol=3;table=1;up=0#sorted_table)  [**size increase (h^-1^)**](http://polywiki.tamu.edu/wiki/bin/view/PolymenisLab/AgingElutriationExperiments?sortcol=3;table=1;up=0#sorted_table)**)** | [**Critical Size (fL)**](http://polywiki.tamu.edu/wiki/bin/view/PolymenisLab/AgingElutriationExperiments?sortcol=2;table=1;up=0#sorted_table) | [**Estimated**](http://polywiki.tamu.edu/wiki/bin/view/PolymenisLab/AgingElutriationExperiments?sortcol=5;table=1;up=0#sorted_table)  [**G1 (T_G1_, h)**](http://polywiki.tamu.edu/wiki/bin/view/PolymenisLab/AgingElutriationExperiments?sortcol=5;table=1;up=0#sorted_table) |
| --- | --- | --- | --- | --- |
| **LL** |  |  |  |  |
| *idh2Δ* | 39.68±2.38 ^b^ | **0.221±0.041^c^** | 62.24±3.64 | 1.72^d^ |
| *hxk2Δ* | **25.93±2.30** | 0.282±0.011 | 65.45±1.66 | 3.06 |
| *lpd1Δ* | **28.86±2.86** | **0.139±0.010** | **54.41±2.21** | 4.5 |
| *tor1Δ* | 43.15±0.14 | **0.248±0.008** | 65.11±3.53 | 1.53 |
| *sch9Δ* | **35.85±0.99** | **0.233±0.020** | 63.24±1.00 | 2.32 |
| *ubr2Δ* | **35.78±2.69** | 0.292±0.016 | **58.00±1.16** | 1.73 |
| *sgf73Δ* | **36.51±1.01** | **0.240±0.016** | **55.28±0.33** | 1.73 |
| *rpp2bΔ* | 40.64±4.09 | 0.299±0.025 | 62.77±1.50 | 1.35 |
| *rei1Δ* | **35.33±2.12** | **0.194±0.024** | 61.2±1.90 | 2.86 |
| *tat2∆* | **37.31±2.01** | 0.301±0.015 | 61.22±2.07 | 1.65 |
| *spt4Δ* | **34.75±1.20** | **0.237±0.017** | 58.73±1.39 | 2.41 |
| *rpl22aΔ* | **32.37±1.31** | **0.219±0.022** | 63.08±2.29 | 2.93 |
| *rpl29Δ* | **37.86±1.54** | **0.231±0.022** | 59.90±1.10 | 2.1 |
| *rpl20bΔ* | **32.80±0.72** | **0.262±0.005** | 60.56±0.97 | 2.25 |
| **NLL** |  |  |  |  |
| BY4743^e^ | 42.12±1.23 | 0.282±0.013 | 61.53±.78 | 1.35 |
| *rps0bΔ^e^* | **34.53±1.89** | **0.238±0.008** | **70.06±1.90** | 2.88 |
| *ylr454wΔ* | 43.87±2.41 | 0.289±0.017 | 61.32±0.98 | 1.35 |
| *tda1Δ* | 41.31±4.95 | 0.282±0.022 | 60.41±1.50 | 1.35 |
| *yal054cΔ* | 40.40±4.09 | 0.285±0.007 | 61.49±1.34 | 1.35 |
| *his7Δ* | 42.52±1.01 | 0.280±0.005 | 61.96±3.08 | 1.35 |
| *rps27bΔ* | 44.29±0.97 | 0.272±0.022 | 62.79±0.71 | 1.35 |
| *sfp1Δ^e^* | **16.04±0.62** | **0.145±0.013** | **39.23±0.0.53** | 6.17 |
| *rpl7bΔ* | 43.05±1.70 | 0.264±0.016 | 60.90±2.10 | 1.35 |
| *rpl34aΔ* | 42.60±0.93 | 0.276±0.038 | 59.94±1.07 | 1.35 |
| *gpa1Δ* | **24.86±0.81** | **0.199±0.008** | **39.10±1.68** | 2.28 |
| *adk1Δ* | **65.29±1.09** | **0.210±0.009** | **102.85±5.73** | 2.1 |
| *dpb4Δ^f^* | **49.92±0.89** | **0.316±0.016** | **64.38±1.08** | 0.82 |

^a^All the strains were in the homozygous diploid BY4743 background, and they were examined in YPD (2% Dextrose) medium. All the strains were examined in at least 3 independent experiments, and in each experiment a technical duplicate was evaluated.

^b^The average and standard deviation are shown in each case.

^c^Values shown in bold differ significantly from the same value of the wild type reference strain (p<0.05; unpaired, 2-tail *t* test, assuming unequal variance). The p values associated with each test were calculated with the corresponding function in Microsoft Excel.

^d^These are G1 estimates from the formula: G1(hours)=Ln(Critical size/Birth size)/*k*, assuming an exponential mode of growth. Note that these values reflect the G1 length of newborn daughter cells. For G1 length calculations, the errors (± sd) were not propagated, and any parameter that was not significantly different from the same parameter of the wild type reference strain was assigned the wild type value.

^e^The values of these strains were obtained from [[48](#_ENREF_48)].

^f^The values of this strain were obtained from previously published values (see Hoose SA, Trinh JT, Leitch MC, Kelly MM, McCormick RF, et al. (2013) Saccharomyces cerevisiae deletion strains with complex DNA content profiles. FEMS Microbiol Lett 345: 72-76.)
