# Supplementary material for: Enhanced Longevity by Ibuprofen, Conserved in Multiple Species, Occurs in Yeast through Inhibition of Tryptophan Import
Source: PLoS Genet. 2014 Dec 18;10(12):e1004860. doi: 10.1371/journal.pgen.1004860 (PMC4270464; doi:10.1371/journal.pgen.1004860)
Supplement: S5 Table — Intracellular amino acid levels in hxk2Δ and sch9Δ cells. (DOCX) [file pgen.1004860.s015.docx]

**Table S5. Intracellular amino acid levels in *hxk2Δ* and *sch9Δ* cells**

|  | ***hxk2Δ*^a^** | | ***sch9Δ*^b^** |  |
| --- | --- | --- | --- | --- |
| **AA** | **[mM]^c^** | **%change^d^** | **[mM]^e^** | **%change^d^** |
| Asp | 6.98±1.90 | -33 | 6.97±5.10 | -33 |
| Glu | 440.13±11.22 | -19 | 341.39±31.08 | -38 |
| Asn | 8.72±7.09 | -57 | 7.11±5.85 | -65 |
| Ser | 71.39±7.03 | -40 | 67.83±7.87 | -43 |
| Gln | 70.73±4.23 | 3 | 45.51±2.85 | -34 |
| His | 34.74±1.22 | -32 | 44.52±11.74 | -14 |
| Gly | 49.46±7.66 | -52 | 44.94±8.37 | -57 |
| Thr | 84.16±24.49 | -15 | 69.42±26.66 | -30 |
| Ala | 44.18±13.91 | -54 | 51.12±5.14 | -47 |
| Arg | 214.78±23.29 | -21 | 264.15±26.69 | -3 |
| Tyr | 5.89±0.84 | -28 | 5.67±1.42 | -30 |
| Val | 31.78±3.28 | -33 | 31.09±7.76 | -34 |
| Met | 8.37±0.81 | -33 | 7.35±0.96 | -40 |
| Trp | 1.34±0.32 | -25 | 1.22±0.17 | -32 |
| Phe | 17.63±2.06 | -39 | 16.49±2.49 | -43 |
| Ile | 21.48±2.89 | -38 | 19.33±3.63 | -45 |
| Leu | 41.99±1.97 | -17 | 41.25±11.36 | -18 |

^a,b^Cells were cultured and harvested as described in Materials and Methods, and they were in the BY4741 background.

^c^The concentrations shown were calculated from the fmoles/cell values we obtained for the analysis, assuming a mean cell size of 35 fL for *hxk2Δ* cells [[83](#_ENREF_83)].

^d^The relative concentration of each amino acid was calculated from the wild type values shown in Table S3, assuming a mean cell size of 45 fL for wild type cells, as we published previously (see Soma S, Yang K, Morales MI, Polymenis M (2014) Multiple metabolic requirements for size homeostasis and initiation of division in Saccharomyces cerevisiae. Microbial Cell 1: 256-266).

^e^The concentrations shown were calculated from the fmoles/cell values we obtained for the analysis, assuming a mean cell size of 43 fL for *sch9Δ* cells [[83](#_ENREF_83)].
