# Supplementary material for: Enhanced Longevity by Ibuprofen, Conserved in Multiple Species, Occurs in Yeast through Inhibition of Tryptophan Import
Source: PLoS Genet. 2014 Dec 18;10(12):e1004860. doi: 10.1371/journal.pgen.1004860 (PMC4270464; doi:10.1371/journal.pgen.1004860)
Supplement: S6 Table — S. cerevisiae strains used in this study. (DOCX) [file pgen.1004860.s016.docx]

**Table S6. *S. cerevisiae* strains used in this study**

| **Strain** | **Genotype** | **Source** |
| --- | --- | --- |
| BY4743 | MATa/α *his3Δ1*/*his3Δ1 leu2Δ0*/*leu2Δ0 lys2Δ0*/*LYS2 MET15*/*met15Δ0 ura3Δ0*/*ura3Δ0* | Open Biosystems |
| BY4741 | MATa *his3Δ1 leu2Δ0 met15Δ0 ura3Δ0* | Open Biosystems |
| BY4742 | MATα *his3Δ1 leu2Δ0 lys2Δ0 ura3Δ0* | Open Biosystems |
| 202233243 | *TAT2-TAP::HIS3MX6* (BY4741 otherwise) | Open Biosystems |
| 202233073 | *NPR1-TAP::HIS3MX6* (BY4741 otherwise) | Open Biosystems |
| 202230811 | *GLN3-TAP::HIS3MX6* (BY4741 otherwise) | Open Biosystems |
| JK9-3dα | MATα *leu2-3,112 ura3-52 trp1 his4 rme1* | Michael N. Hall |
| JH11-1c | *TOR1-1* (JK9-3dα otherwise) | Michael N. Hall |
| JH12-17b | *TOR2-1* (JK9-3dα otherwise) | Michael N. Hall |
| CHY01 | *tat1Δ::KANMX4, tat2::KANMX4* (BY4742 otherwise) | This study |
| CHY02 | *tat2∆::URA3* (BY4742 otherwise) | This study |
| CHY03 | *2HA-TAT2* (BY4742 otherwise) | This study |
| CHY04 | *2HA-5KR-TAT2* (BY4742 otherwise) | This study |
| CHY05 | *hxk2Δ::URA3* (CHY04 otherwise) | This study |
| CHY06 | *rpl20bΔ::URA3* (CHY04 otherwise) | This study |
| CHY07 | *hxk2Δ::URA3* (202233243 otherwise) | This study |
| CHY08 | *rpl20bΔ::URA3* (202233243 otherwise) | This study |
| CHY09 | *sch9Δ::URA3* (202233243 otherwise) | This study |
