# Supplementary material for: Enhanced Longevity by Ibuprofen, Conserved in Multiple Species, Occurs in Yeast through Inhibition of Tryptophan Import
Source: PLoS Genet. 2014 Dec 18;10(12):e1004860. doi: 10.1371/journal.pgen.1004860 (PMC4270464; doi:10.1371/journal.pgen.1004860)
Supplement: S7 Table — Primer pairs for quantitative real-time PCR. (DOCX) [file pgen.1004860.s017.docx]

**Table S7. Primer pairs for quantitative real-time PCR**

| **Gene** | **Orientation** | **Primer Sequence (5’ to 3’)** |
| --- | --- | --- |
| *TAT1* | Forward | CAGTTTCATTGCCAAAGAGG |
| *TAT1* | Reverse | GTACCGAGACTGATCATGAC |
| *TAT2* | Forward | CTGTCAAGCGTTCAAATGAG |
| *TAT2* | Reverse | GGCAATCATGATTAAGTGCC |
| *GAP1* | Forward | GAGTTTCTAACTCAGGAGCC |
| *GAP1* | Reverse | GTCTATTCTTCAAGTGGTGC |
| *GLN1* | Forward | GAACTGGACCAAAGAGGTAG |
| *GLN1* | Reverse | GGATCTGGGTAGTAAGCAAC |
| *PUT1* | Forward | GTTACTAAATCGCGCATACC |
| *PUT1* | Reverse | CCAGTTGGAATAGTTCCTTC |
| *PUT4* | Forward | CCCTTCCACAAGAACAATAG |
| *PUT4* | Reverse | GATTTCTCCAAGTCCACGTC |
| *DAL4* | Forward | CAGTAATCCTTCGAGGAAAG |
| *DAL4* | Reverse | CTCCAGATGGACTTTGTTTC |
| *DAL5* | Forward | CTTCAGAAGCTGAAATCAAG |
| *DAL5* | Reverse | CAATAGTCGATCTTCCAACG |
| *HIS4* | Forward | GTCAGGTACTTTTGGATGGC |
| *HIS4* | Reverse | CCGTTCTCTTCCACAACAAC |
| *ARG1* | Forward | GTGGTTTAGATACCTCCGTC |
| *ARG1* | Reverse | GGCTTTGGCAATAACAGGTC |
| *ARO9* | Forward | CAACCGTTTCTCTCCAGAAG |
| *ARO9* | Reverse | CCTTGGTTTCTGCATATTGG |
| *ARO10* | Forward | CCTAGTGTTGAATCAGCTGG |
| *ARO10* | Reverse | GACATTTTCAGCGAACGAAC |
